# Photoinduced elimination of senescent microglia cells *in vivo* by chiral gold nanoparticles†

**DOI:** 10.1039/d2sc01662a

**Published:** 2022-05-16

**Authors:** Zhuojia Xu, Aihua Qu, Hongyu Zhang, Weiwei Wang, Changlong Hao, Meiru Lu, Baimei Shi, Liguang Xu, Maozhong Sun, Chuanlai Xu, Hua Kuang

**Affiliations:** International Joint Research Laboratory for Biointerface and Biodetection, State Key Lab of Food Science and Technology, School of Food Science and Technology, Jiangnan University Wuxi Jiangsu 214122 People's Republic of China smz@jiangnan.edu.cn xcl@jiangnan.edu.cn

## Abstract

Parkinson's disease (PD) is an age-related neurodegenerative disease, and the removal of senescent cells has been proved to be beneficial for improving age-associated pathologies in neurodegeneration disease. In this study, chiral gold nanoparticles (NPs) with different helical directions were synthesized to selectively induce the apoptosis of senescent cells under light illumination. By modifying anti-B2MG and anti-DCR2 antibodies, senescent microglia cells could be cleared by chiral NPs without damaging the activities of normal cells under illumination. Notably, l-P^+^ NPs exhibited about a 2-fold higher elimination efficiency than d-P^−^ NPs for senescent microglia cells. Mechanistic studies revealed that the clearance of senescent cells was mediated by the activation of the Fas signaling pathway. The *in vivo* injection of chiral NPs successfully confirmed that the elimination of senescent microglia cells in the brain could further alleviate the symptoms of PD mice in which the alpha-synuclein (α-syn) in cerebrospinal fluid (CFS) decreased from 83.83 ± 4.76 ng mL^−1^ to 8.66 ± 1.79 ng mL^−1^ after two months of treatment. Our findings suggest a potential strategy to selectively eliminate senescent cells using chiral nanomaterials and offer a promising strategy for alleviating PD.

## Abstract 2

The apoptosis pathways of senescent microglia cells induced by chiral NPs under the irradiation of 808 nm laser in the brain of PD mice.

## Graphical abstract

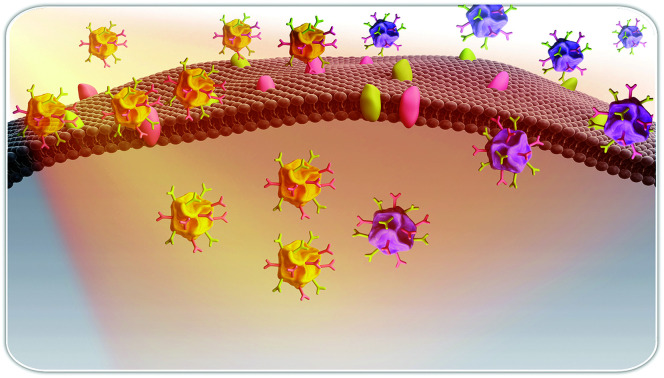

## Introduction

Parkinson's disease (PD) is an age-related brain disease that is associated with motivation and cognitive disorders and the assembly of alpha-synuclein (α-syn); there is no effective therapeutic treatments for this condition.^1^ Pathological changes associated with PD are related to an increase in reactive oxygen species,^2^ the inappropriate folding of α-syn,^3–6^ homeostasis disorders and inflammation in the main neuropathological area of the brain.^7–11^ Along with tissue dysfunction, the typical senescence-associated secretory phenotype is significantly characterized by the generation of interleukin-6 (IL*-*6) and interleukin-1β (IL*-*1β).^12,13^ Indeed, the accumulation of senescent cells is associated with a range of age-related diseases as well as neurodegenerative diseases.^12,14–19^ However, it is still unclear how senescence in the brain contributes to PD and what role it might play in therapeutic strategies of PD. It had been reported that senescent cells could give rise to local and systemic inflammation and contribute to neurodegeneration in neurodegeneration diseases like PD.^20–22^ In addition, direct exposure to Aβ was shown to cause senescence in oligodendrocyte precursor cells (OPCs), and the clearance of OPCs by senolytic therapy alleviated Aβ-associated inflammation and restored cognitive deficits in AD mice, thus illustrating the potential for senescence clearance to be used in clinical practice.^23^ Moreover, senescent cells play a role in the initiation and progression of tau-mediated disease, and targeting of senescent cells may provide a therapeutic avenue for the treatment of such pathologies.^24^ Therefore, eliminating senescent cells may hold therapeutic promise for alleviating the symptoms of PD.^25^

Inorganic nanomaterials that exhibit chirality exist commonly in nature and have attracted significant attention due to their promising properties and potential for multi-biological applications related to the adjustment of life progression.^26–28^ The different interactions of chiral nanoparticles (NPs) with bio-interfaces can induce a range of biological reactions.^27,29,30^ Our group has made several key breakthroughs in this field. For example, we found that the maturation of immune cells can be regulated by nanoscale chirality *via* differential affinities for biological receptors.^31^ Moreover, due to their biocompatibility and easily controllable optical activity, chiral NPs have already been applied for the elimination of senescent cells and the restoration of homeostasis.^13,32^ Light provides a non-invasive method for controlling these light-responsive nanomaterials due to its precise spatial resolution, appropriate controllability and the lack of negative effects in these light-responded biomimetic systems consisting of nanomaterials.^26,33–35^ Alternatively, a recent study has demonstrated that one biomimetic NP could effectively target and modulate microglia cells and thus be expected to be a potential medicine against PD.^36^ Collectively, the evidence provides the possibility of alleviating neurodegenerative disease by the application of chiral NPs to clear senescent microglia cells.^37–39^

In the present study, we prepared chiral NPs with high anisotropy factors to eliminate senescent microglia cells from the brain under NIR irradiation at 808 nm. We found that l-P^+^ NPs exhibited the maximum clearance efficiency of senescent cells without damaging normal cells. Senescent microglia cells were eliminated by the activation of an apoptotic pathway by mechanical force under light; this was demonstrated by the increased expression of apoptotic genes in the treatment groups. We also used a mouse model of PD to evaluate this potential therapeutic strategy *in vivo*. Following two months of treatment, the pathological symptoms of the PD mice were greatly improved with the decreased concentration of α-syn and the repair in motor ability.

## Results and discussion

### Surface modification of chiral NPs and construction of the model of senescence

**Fig. 1:**
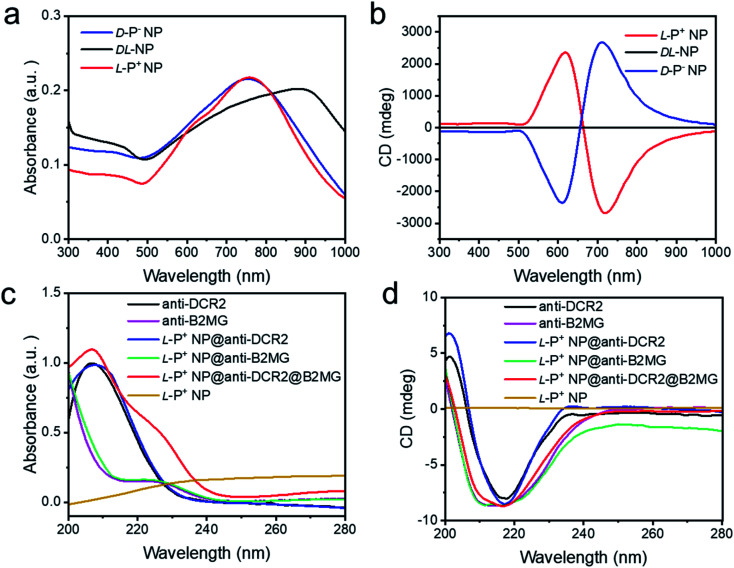
The (a) UV-Vis absorption and (b) CD spectra of chiral NPs. The (c) UV-Vis absorption and (d) CD spectra of anti-DCR2, anti-B2MG, l-P^+^ NP@anti-DCR2, l-P^+^ NP@anti-B2MG, l-P^+^ NP@anti-DCR2@anti-B2MG and l-P^+^ NP in the 200–280 nm range.

**Scheme 1:**
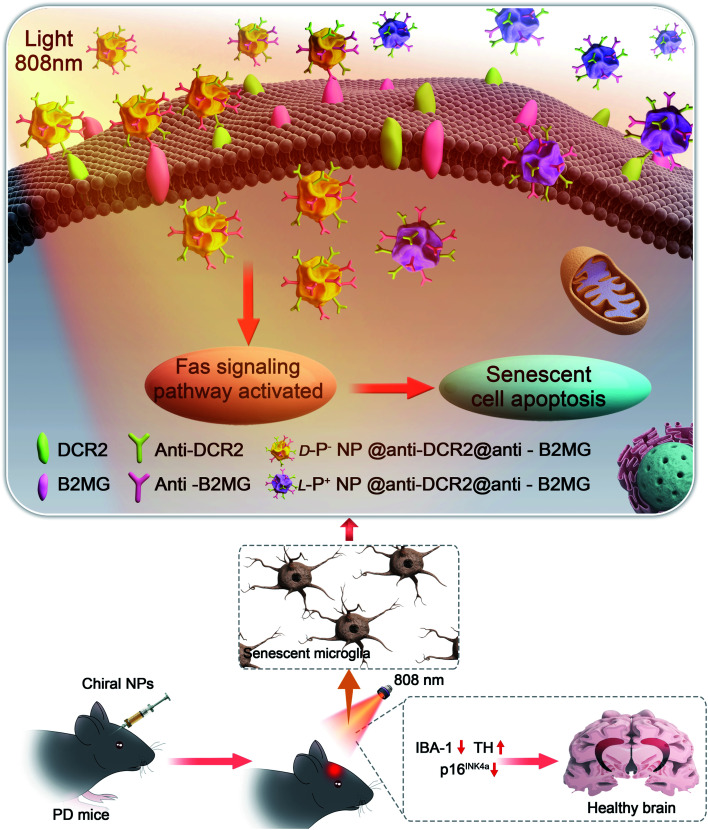
Illustration of the apoptosis pathways of senescent microglia cells induced by chiral NPs under the irradiation of 808 nm laser in the brain of PD mice.

Chiral NPs (denoted as l-P^+^ NP, d-P^−^ NP, and dl-NP) of different handedness were synthesized in accordance with a previous study using l-, d-, and dl-Cys-Phe (CF) as the chiral ligands.^31^ As shown in Fig. S1a and b,† the scanning electron microscopy images showed an obvious chiral helical direction of nanomaterials. The dl-NPs with no chiral morphology are shown in Fig. S1c.† The absorption and circular dichroism (CD) spectra were used to illustrate the strong optical signals of the three types of chiral NPs in the visible and NIR range (Fig. 1a and b). In addition, the anisotropy factor (*g*-factor) of l-P^+^ NPs reached 0.44 at 711 nm, while that of d-P^−^ NP reached 0.42 at 722 nm, and that of dl-NP was 0 (Fig. S2†). To selectively recognize senescent cells, the chiral NPs were modified with anti-B2MG and anti-DCR2 antibodies, two established markers of senescent cells.^32,33,40^ The successful conjugation of antibodies with chiral NPs was demonstrated by the absorbance and CD spectrum being in the 200–280 nm range, in which a new peak created by the conjunction of the NPs and the antibodies appeared at 210–240 nm (Fig. 1c and d). Moreover, the CD spectrum illustrated that there was no influence on the chirality of nanomaterials with antibodies coated (Scheme 1 and Fig. S3†).

**Fig. 2:**
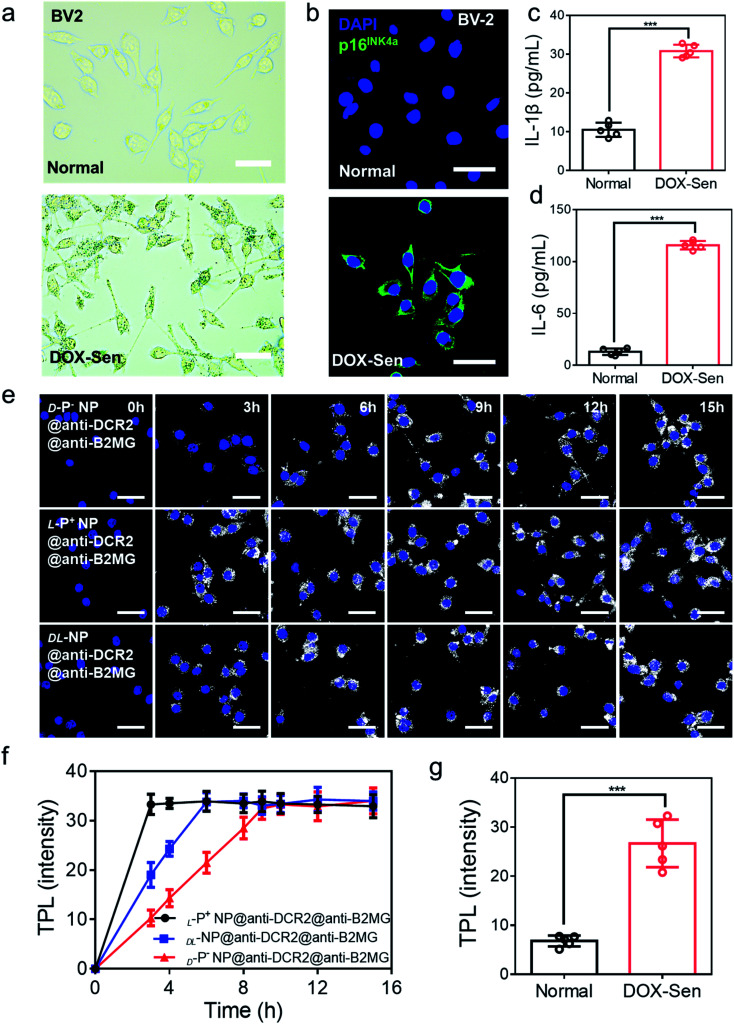
(a) The β*-*Gal staining assay and (b) confocal images of BV-2 cells before (up) and after (down) treatment with 10 nM DOX for 7 days. Scale bars, 50 μm. Blue, 4′,6-diamidino-2-phenylindole (DAPI) for nuclei; green, p16^INK4a^, the marker of senescent cells. Scale bars, 30 μm. (c) The IL-1β and (d) IL-6 expression in normal cells and DOX-induced senescent BV-2 cells were quantified by ELISA. (e) TPL images of senescent BV-2 cells incubated with chiral NPs for different times respectively. Scale bars, 30 μm. (f) The statistic intensity from the TPL images in (e) and Fig. S8.† (g) The statistic TPL intensity of normal cells and senescent cells incubated with l-P^+^ NP@anti-B2MG@anti-DCR2 in Fig. S9.† Data are presented as mean ± s.d. (*n* = 5). ***p < 0.001.

A previous study had illustrated that microglia cells are associated with the loss of dopamine neurons, which was considered as one of the main reasons for PD.^2,22^ Therefore, BV-2 cells were incubated with 10 nM doxorubicin (DOX) for 7 days to induce senescent models (DOX-Sen) to monitor the pathological symptoms in the brains affected by PD.^33^ After incubation with DOX, the increased expression of β*-*galactosidase (β*-*Gal) and up-regulated expression of p16^INK4a^ (a known marker of senescence)^13^ confirmed the successful construction of the senescence models *in vitro* (Fig. 2a and b). In addition, as shown in Fig. 2c and d, the concentrations of IL*-*1β and IL*-*6 were measured by enzyme-linked immunosorbent assay (ELISA) to indicate the state of inflammation in the senescent cells. When incubated with DOX, the BV-2 cells showed signs of senescence with increased expression levels of inflammation markers.

Then, the appropriate concentrations of anti-DCR2 and anti-B2MG binding to the NPs were optimized under two-photon luminescence (TPL) after incubating the chiral NPs with senescent BV-2 cells. As shown in Fig. S4 and S5,† the maximum TPL intensity was obtained when 1 nM anti-B2MG and 0.8 nM anti-DCR2 were attached to the l-P^+^ NPs. Therefore, we used 0.8 nM of anti-DCR2 and 1 nM of anti-B2MG for the subsequent experiments as we had optimized in experimental cells. In addition, to evaluate the difference of nanomaterials with different chiral morphologies in binding antibodies, the CD spectrum in 200–280 nm was collected. As shown in Fig. S6,† it could be concluded from the CD spectrum that there was no obvious disparity in l-P^+^ NP, d-P^−^ NP, and dl-NP after removing the dissociative antibodies.

We used cell counting kit-8 (CCK-8) experiments to monitor the cytotoxicity of chiral NPs in senescent cells (Fig. S7†). We found that the concentration of 800 μg mL^−1^ exerted no obvious cytotoxic effects on the three types of senescent cells. Furthermore, to illustrate the chiral effect of NPs on senescent cells, we investigated the uptake ability of senescent BV-2 cells for chiral NPs. The relationship between the incubation time and the amount of intracellular chiral NPs was determined from the TPL intensity (Fig. 2e and S8†). As shown in Fig. 2f, l-P^+^ NPs reached maximal values after 3, 6, and 6 h in senescent BV-2 cells. By contrast, d-P^−^ NP reached maximum values after 9, 12, and 12 h in senescent BV-2 cells. These data indicated the higher uptake efficiency of l-P^+^ NP for senescent BV-2 cells which was 3.07-fold higher than that of d-P^−^ NPs. The TPL intensity showed limited endocytosis in normal cells and further demonstrated the specific recognition of senescent cells (Fig. 2g and S9†). Moreover, to make out how antibodies and the chiral morphology affect the endocytosis of nanomaterials, we considered several groups (senescent cells: l-P^+^ NP, d-P^−^ NP, dl-NP; normal cell: l-P^+^ NP, d-P^−^ NP, dl-NP; senescent cells: l-P^+^ NP@anti-DCR2@anti-B2MG, d-P^−^ NP@anti-DCR2@anti-B2MG, dl-NP@anti-DCR2@anti-B2MG; normal cells: l-P^+^ NP@anti-DCR2@anti-B2MG, d-P^−^ NP@anti-DCR2@anti-B2MG, dl-NP@anti-DCR2@anti-B2MG; normal cells) to clarify it (Fig. S10 to S14†). It is showed that the nanomaterials coated with antibodies had higher efficiency in senescent cells than in normal cells, which was about 3-fold. Notably, we found that in both normal cells and senescent cells, l-P^+^ NPs exhibited a higher uptake efficiency at about 3-fold higher than that of d-P^−^ NPs whether coated with or without antibodies.

To further explore the endocytosis pathway by which l-P^+^ NPs with or without antibodies coated were taken in different cells, normal and senescent BV-2 cells were incubated with different inhibitors of endocytosis pathways.^31^ As shown in Fig. S15 and S16,† the uptake of l-P^+^ NP@anti-DCR2@anti-B2MG in both normal and senescent BV-2 cells was inhibited when the pathways of clathrin and dynamin were blocked respectively. Simultaneously, the uptake of l-P^+^ NP in normal and senescent cells was studied (Fig. S17 and S18†). When the pathways of clathrin and dynamin were blocked, few l-P^+^ NPs were taken up by both senescent and normal cells. It showed that there was no difference in the endocytosis pathway by which l-P^+^ NPs with or without antibodies coated were taken in both normal and senescent cells (Fig. S19 and S20†). These results further prove the orienting function of antibodies and highlight the role of chirality in endocytosis.

### Light-induced apoptosis of senescent cells caused by chiral NPs

**Fig. 3:**
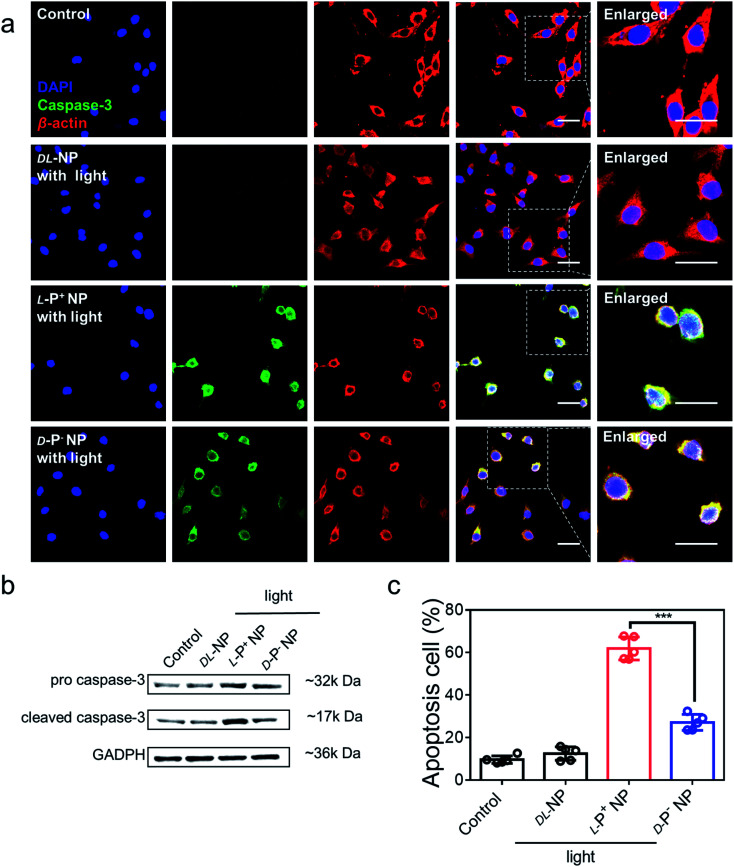
(a) Confocal images of senescent BV-2 cells incubated with NPs coated with antibodies under light. Senescent BV-2 cells without any treatment were set as the control. Red, β*-*actin; green, caspase-3; blue, DAPI for nuclei. Scale bars, 40 μm. (b) Western blot analysis for pro caspase-3 and cleaved caspase-3 in senescent BV-2 cells, GAPDH was set as the control. (c) The apoptosis of senescent BV-2 cells with different treatments measured by flow cytometry. Data are presented as mean ± s.d. (*n* = 5). ***p < 0.001.

The light-responsive ability of chiral NPs was studied using illumination provided by an 808 nm laser according to absorption. The illumination power and time were optimized to minimize the photothermal effects that may damage living cells. All chiral nanomaterials used in the following experiments were modified with antibodies unless otherwise specified. As shown in Fig. S21,† after 5 minutes of illumination at 400 mW cm^−2^, the temperature of the l-P^+^ NPs showed only a minimal increase in living cells with incubating with NPs for 3 h; these conditions were therefore selected for further experiments. To investigate the ability of the chiral NPs to clear senescent cells, the representative apoptotic protein caspase-3 was monitored. We used confocal imaging to identify cellular apoptosis; the fluorescence intensity of caspase-3 in apoptotic cells increased. Under irradiation with a laser at 808 nm, the expression of the apoptosis-related protein caspases-3 reached maximum levels after incubation with l-P^+^ NPs for 6 h when compared with groups treated with d-P^−^ and dl-NPs (Fig. 3a). As shown in Fig. S22,† it was clear that the senescent microglia cells were eliminated *via* the apoptotic pathway and l-P^+^ NPs exhibited higher efficiency than the other types. In addition, we used western blot analysis to investigate the expression of cleaved caspases-3 in cells under different treatments (Fig. 3b and S23†). We also used flow cytometry to further demonstrate the levels of apoptosis in senescent cells induced by chiral NPs under light. As shown in Fig. 3c, the proportion of apoptotic cells in senescent microglia cells treated with l-P^+^ NPs under light reached 61.89% ± 5.85%; this was almost 2.28-fold higher than that of the groups treated with d-P^−^ NPs. Typical flow cytometry scatter plots representing apoptosis of cells are shown in Fig. S24.†

As a control, only minimal levels of caspases-3 were detected in senescent cells after incubation with chiral NPs coated with antibodies but without illumination, which had no significant difference with the control (Fig. S25†). This result indicated that there was no significant apoptosis in the senescent cells if irradiation was not provided (Fig. S26†). Furthermore, when chiral NPs (without anti-DCR2 and anti-B2MG modification) were incubated with senescent cells for 6 h, only minimal levels of the caspase-3 expression were present under illumination (Fig. S27 and S28†). Moreover, we investigated the clearance efficiency of chiral NPs coated with or without anti-B2MG and anti-DCR2 when exposed to normal cells under irradiation. As shown in Fig. S29 and S30,† after incubation with l-P^+^ NPs under illumination for 10 min, the experimental groups showed the same expression levels around 10% with untreated normal cells, thus indicating that the chiral NPs did not induce side effects in normal cells.

These results suggest that the targeting clearance ability towards senescent cells was mediated through the apoptotic pathway after incubation with chiral NPs modified by anti-DCR2 and anti-B2MG under illumination. Moreover, l-P^+^ NPs exhibited a higher clearance efficiency of senescent cells than the other types.

### The mechanism underlying the induction of apoptosis in senescent microglia cells by chiral NPs under light

**Fig. 4:**
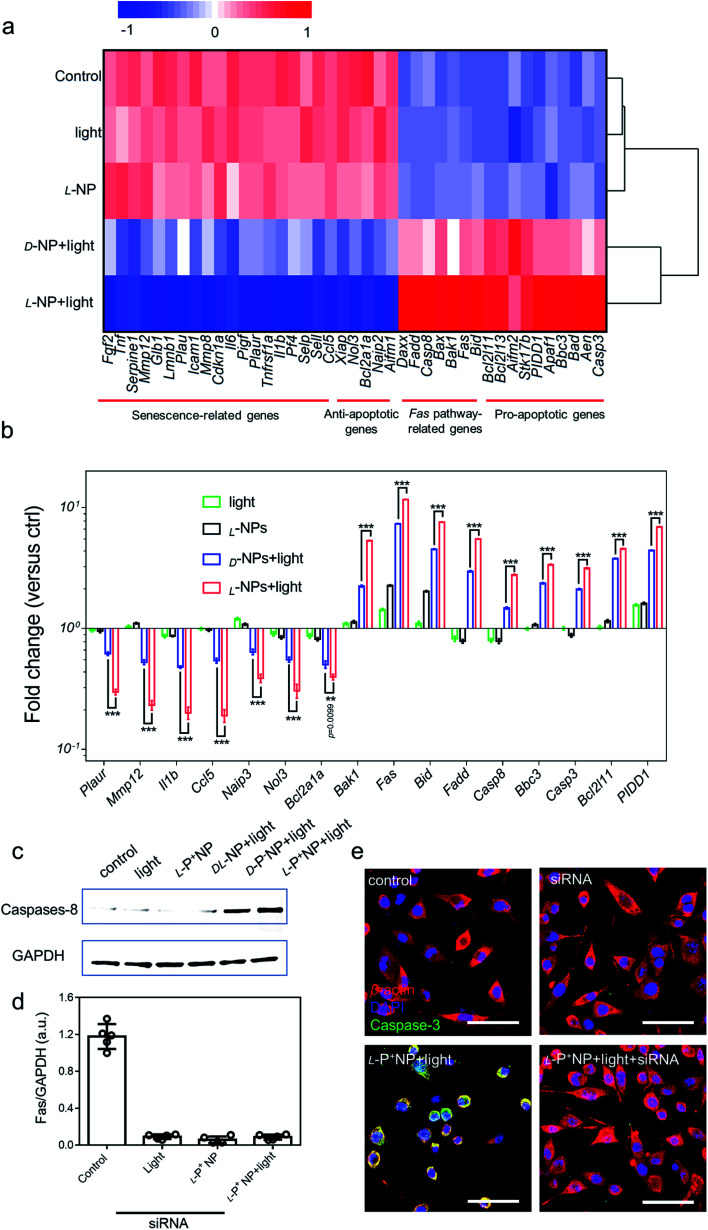
(a) Heatmap of the senescence-related, anti-apoptotic, Fas pathway-related and pro-apoptotic gene expressions in BV-2 cells with different treatments. Senescent BV-2 cells with no treatment was set as the control. (b) The differentially expressed genes from the heatmap results are expressed as fold changes compared with levels in the control. (c) Western blot analysis for cleaved caspase-8 in senescent BV-2 cells with different treatments; GAPDH was set as the control. (d) Results of western blot analysis for Fas in senescent BV-2 cells with different treatments in Fig. S32.† (e) Confocal images of senescent BV-2 cells with different treatments: siRNA for Fas; l-P^+^ NP and l-P^+^ NP + light + siRNA. Red, β*-*actin; green, caspase-3; blue, DAPI for nuclei. Scale bars, 60 μm. Data are presented as mean ± s.d. (*n* = 5). ***p < 0.001.

Next, we investigated the global expression profiles of microglia cells to determine the mechanisms responsible for the induction of apoptosis in senescent cells by chiral NPs under light (Fig. 4a). There were clear differences between the experimental (senescent BV-2 cells treated with: light, l-P^+^ NP; d-P^−^ NP + light; l-P^+^ NP + light) and control groups (senescent BV-2 cells without any treatment), as summarized in Fig. 4b; our results indicated that the expression of senescence-related genes (*Plaur*, *Mmp12*, *Il1b*, *Ccl5*)^7,15,16^ was significantly reduced in senescent BV-2 cells after treatment with l-P^+^ NP under light. Moreover, as compared with the control group, the expression levels of anti-apoptotic genes (*Aifm1*, *Xiap*, *Ccl5*, *Nol3* and *Naip2*)^13^ were decreased while the levels of proapoptotic genes (*Aifm2*, *Aen*, *Bbc3*, *PIDD1* and *Bcl2l11*) were increased in senescent BV-2 cells by l-P^+^ NPs under light.^41^ Importantly, we found that in the senescent microglia cells treated with l-P^+^ NPs under light, the expression levels of Fas signaling pathway-associated genes (*Casp8*, *Fadd*, *Bid* and *Fas*),^42,43^ an outcome mediated by the activation of the apoptosis pathway, were higher than in any of the other groups. These data indicated that the apoptosis of senescent cells induced by l-P^+^ NPs was through the Fas signaling pathway.

To confirm the activation of the Fas signaling pathway,^44–46^ the expression of cleaved caspase-8, the key protein in this pathway, was measured. As shown in Fig. 4c, the expression of cleaved caspase-8 in d-/l-NP and light treated cells was higher than that in the control, of which the expression in l-P^+^ NPs was 22.09-fold higher than that in the control and that of d-P^−^ NPs was 12.21-fold higher. This result clearly confirmed that apoptosis in chiral material treated groups was mediated by the Fas signaling pathway. Notably, the expression of caspase-8 in the group treated with l-P^+^ NPs and light was 1.81-fold higher than that with d-P^−^ NPs and light (Fig. S31†). Moreover, small interfering RNA (siRNA) that specifically targeted Fas was designed to knockout the expression of Fas.^47^ During incubation with siRNA, the expression of Fas was successfully inhibited by western blot analysis (Fig. 4d). In the siRNA-treated groups, the expression of cleaved caspase-8 was dramatically inhibited in the group treated with l-P^+^ NPs, similar to the cells with no treatments (Fig. S32†). In addition, confocal images also showed the decreased expression of caspase-3 from 51.18 to 11.29 in l-P^+^ NPs under light group after siRNA treatment (Fig. 4e and S33†). Taken together, these data indicated that the apoptosis induced by l-P^+^ NPs under light was mediated by the Fas signaling pathway.

### Chiral NPs ameliorated the symptom of senescence in PD mice *in vivo*

The high elimination efficiency of the chiral NPs upon senescent cells and their perfect biocompatibility inspired us to apply these engineered particles for the therapeutic treatment of a mouse model of PD and explored their potential to ameliorate the symptoms of PD by clearing senescent cells.

**Fig. 5:**
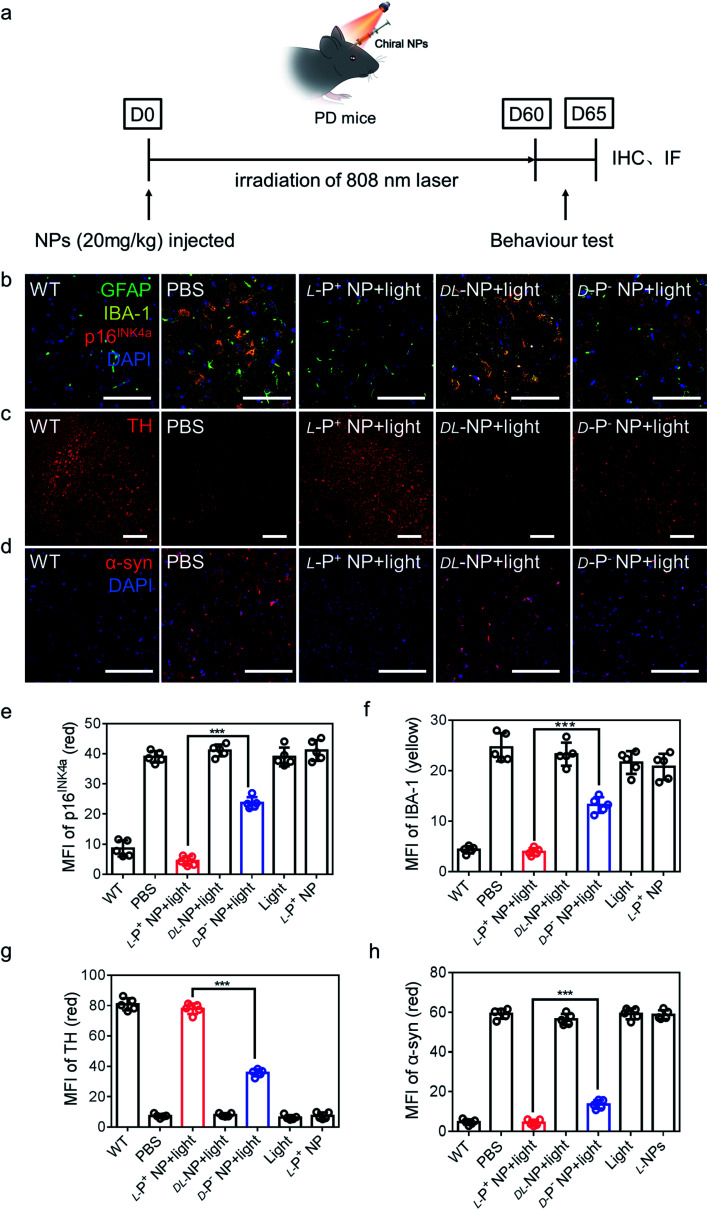
(a) Schematic illustration of l-P^+^ NPs injected into PD mice. (b) Immunofluorescence staining images of GFAP, IBA-1 and p16^INK4a^ in the nigra of different mice. Green, GFAP; yellow, IBA-1; blue, DAPI for nuclei. Scale bars, 60 μm. (c) Immunofluorescence staining images of TH in the nigra of different mices. Scale bars, 50 μm. (d) Immunofluorescence staining images of α-syn in the nigra of different groups. MFI of (e) p16^INK4a^ and (f) IBA-1 in the nigra for the result in (b) and Fig. S46,† MFI of (g) TH in the nigra for the result in (c) and Fig. S47.† MFI of (h) α-syn in the nigra for the result in (d) and Fig. S48.† Scale bars, 100 μm. Data are presented as mean ± s.d. (*n* = 5). ***p < 0.001.

Female PD model mice (A53T) were used for the *in vivo* experiment. Chiral NPs, combined with anti-B2MG and anti-DCR2 (20 mg kg^−1^), were injected into the striatum of each mouse (anteroposterior +0.4 mm, mediolateral ±1.5 mm and dorsoventral −2.88 mm)^4^ (Fig. 5a).

To make out the distribution of chiral materials after injecting into the brain of PD mice (senescent neurons: p16^INK4a+^ and MAP2^+^; senescent astrocytes: p16^INK4a+^ and GFAP^+^; senescent microglia p16^INK4a+^ and IBA-1^+^), l-P^+^ NPs labeled with Cy3 in different brain cells were measured by flow cytometry. As shown in Fig. S34,† the chiral materials were saturated in the brain of PD mice after injection for 12 h and around 97.7% of l-P^+^ NPs were in senescent cells. In addition, 78.2% of the Cy3 fluorescence was detected in senescent microglia cells, which indicated that the l-P^+^ NPs were almost taken up by senescent microglia cells (Fig. S35†).

The mice were then exposed to irradiation at 808 nm under NIR conditions at 600 mW all day for a 2 month period. Moreover, PD mice were injected with PBS and illuminated under the same conditions as controls. H&E staining assays were used to investigate the striatum and nigra from mice undergoing different treatments. These data illustrated that the injection of NPs caused no obvious damage in the brain (Fig. S36†). Furthermore, H&E staining assays of the heart, liver, kidneys, spleen, and lungs of mice under different treatments indicated the excellent biocompatibility and low biotoxicity of the chiral NPs (Fig. S37†). Furthermore, we evaluated the impacts of l-P^+^ NPs on blood chemistry and the accumulation of l-P^+^ NPs in the major organs after intravenous injection to reveal their *in vivo* biocompatibility. As shown in Fig. S38,† the concentration of liver function indicators (aspartate transaminase (AST) and alanine transaminase (ALT)) and kidney function indicators (BUN and CRE) indicated that there was no difference in the NP-treated group and control, revealing good biocompatibility in the liver and kidneys. Moreover, the liver and kidneys exhibited higher accumulation of NPs and the concentration decreased soon in blood in 48 h, while those in the heart, spleen and lungs were lower, indicating the rapid clearance of l-P^+^ NPs from the body to guarantee excellent biocompatibility (Fig. S39†).

Next, we investigated the motor function of PD mice before and after treatment. Notably, light-exposed PD mice injected with l-P^+^ NPs exhibited a remarkable restoration in the rotarod test and wheeling running test but with an increased latency to fall and a longer calculative distance. The latency of mice treated with l-P^+^ NPs was almost 1.84-fold higher than that for mice treated with d-P^−^ NPs, and the cumulative distance was almost 1.67-fold higher than that of mice treated with d-P^−^ NPs (Fig. S40†). The speed of mice in the wheel running test was also determined. Mice treated with l-P^+^ NPs and light exhibited the same maximum speed as normal mice, thus indicating that the PD mice have recovered their motor ability (Fig. S41†). In addition, the spatial cognition and memory of mice undergoing different treatments were investigated using the Morris water maze. Light-exposed PD mice injected with l-P^+^ NP exhibited a significantly reduced latency for finding the hidden platform; l-P^+^ NPs caused the latency to reduce 84.43% ± 5.13% as compared with the d-P^−^ NP treatment group, thus indicating that a remarkable recovery in the spatial memory disorder of PD mice was obtained after l-P^+^ NP and light treatment (Fig. S42†). Track sheets of the alterations in the locomotion of different mice are shown in Fig. S43.†

Next, we performed the co-localization studies of a neuronal cell marker (Map2), a microglia marker (IBA-1), an astrocyte markers (GFAP), and a senescence marker (p16^INK4a^) in the brain of PD mice. We found that the expression of microglia and p16^INK4a^ overlapped obviously while the expression of markers for neurons and astrocytes did not overlap with senescence markers (Fig. S44 and S45†). In addition, immunofluorescence staining results from brain sections showed that the main senescent cells were microglia cells in the PD model mice and the proportion of senescent microglia decreased to 81.18% ± 6.54% when compared with that of groups treated with d-P^−^ NPs with light (Fig. 5b, e and S46†).

IBA-1, which also reflects the levels of neuroinflammation, decreased to 70.37% ± 3.52% in PD mice after treatment with l-NPs and light when compared with those treated with d-P^−^ NPs under light (Fig. 5f). In addition, there was an increase to 77.908 in the MFI of tyrosine hydroxylase (TH), an enzyme that plays a key role in the synthesis of dopamine (Fig. 5c, g and S47†). These data indicated that treatment with l-P^+^ NPs and light caused a remission of the symptoms of PD.

We also stained brain sections to detect α-syn, the biomarker for PD that reflects the progression of this neurodegeneration disease, and observed a significant reduction to 4.378 in the MFI of α-syn in the group treated with l-P^+^ NPs under light (Fig. 5d, h and S48†). Additionally, to make out the distribution of NPs in brain, IHF images of l-P^+^ NP-Cy3 were obtained. As shown in Fig. S49,† the fluorescence of NPs was mainly detected in the section of nigra, which could be attributed to the senescence of microglia in nigra in PD mice.

**Fig. 6:**
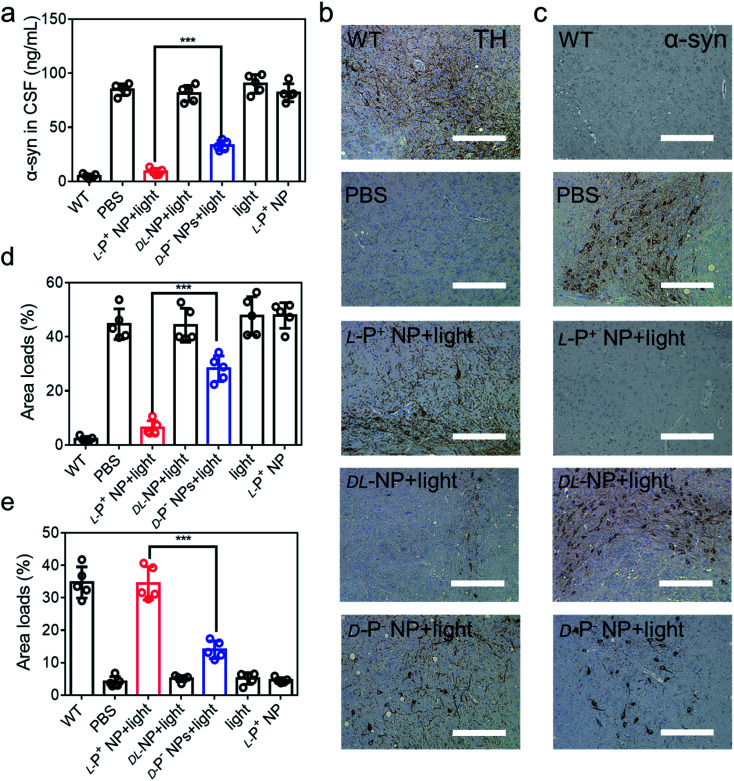
(a) The concentration of α-syn in CSF of mice with different treatments. IHC images of (b) TH and (c) α-syn in the brains (substantia nigra) of different mice. Scale bars, 100 μm. The results of quantitative analysis of (d) TH in (b) and Fig. S50a,† and (e) α-syn loads in (c) and Fig. S51b† in the brains. Data are presented as mean ± s.d. (*n* = 5). ***p < 0.001.

Furthermore, the aggregation of α-syn was detected using ELISA kits (Jiangsu Jingmei Biological Technology Co., Ltd). The reduced amount of α-syn in the treated group was indicative of potential remission of PD after treatment with chiral NPs under irradiation for 60 days. Fig. 6a shows that there was a significant attenuation in the levels of α-syn in PD mice when treated with l-P^+^ NPs and light, which decreased from 83.83 ± 4.76 ng mL^−1^ to 8.66 ± 1.79 ng mL^−1^. Moreover, the levels of α-syn in the group treated with l-P^+^ NPs decreased to 71.69% ± 9.85% when compared with those in the group treated with the d-P^−^ NPs due to the chiral effect of the NPs. In addition, the dl-NP and light treated group has no obvious difference compared to the PD group in all test indices, indicating that NPs with no chirality had no therapy potential of PD, which corresponded with the results *in vitro*.

To further confirm these results, we performed immunohistochemical staining (IHC) to detect TH and α-syn (Fig. 6b, c and S50†). As shown in Fig. 6d and e, the distribution of TH in the brain increased in mice treated with l-P^+^ NPs and light while that of α-syn decreased. Corresponding β-Gal staining images of the nigra also indicated the clearance of senescence in PD mice treated with l-P^+^ NPs under light (Fig. S51†). Collectively, these data indicate that l-P^+^ NPs with light illumination could efficiently clear senescence in the brains of PD mice and thus alleviate the pathological symptoms of PD *in vivo*.

## Conclusion

In conclusion, we successfully fabricated light-responsive chiral gold NPs for the clearance of senescent microglia cells selectively. *In vivo* mice experiments demonstrated that l-P^+^ NPs accumulated in senescent microglia cells in the brain of PD mice and effectively cleared senescent microglia cells under light, which decreased the amount of α-syn in CSF and recovered the dyskinesia of PD mice. This discovery paved the way for the use of chiral nanomaterials for fundamental research in clinical neurodegeneration disease therapy.

## Data availability

All data supporting the findings of this study are available within the article and in the ESI.†

## Author contributions

C. X., H. K., and M. S. conceived the concept and directed the project. Z. X. and A. Q. carried out the experiments and analysed the data. W. W., M. L. and B. S. collected the data. C. H., H. Z. and L. X. discussed the results. Z. X. prepared the first draft of this manuscript, and M. S. and C. X. modified the manuscript. All authors commented on the manuscript.

## Conflicts of interest

The authors declare no competing financial interest.

## Supplementary Material

SC-013-D2SC01662A-s001

## References

[cit1] Bloem B. R., Okun M. S., Klein C. (2021). Parkinson's disease. Lancet.

[cit2] Hao C., Qu A., Xu L., Sun M., Zhang H., Xu C., Kuang H. (2019). Chiral Molecule-mediated Porous Cu_x_O Nanoparticle Clusters with Antioxidation Activity for Ameliorating Parkinson's Disease. J. Am. Chem. Soc..

[cit3] Liu J., Liu C., Zhang J., Zhang Y., Liu K., Song J. X., Sreenivasmurthy S. G., Wang Z., Shi Y., Chu C., Zhang Y., Wu C., Deng X., Liu X., Song J., Zhuang R., Huang S., Zhang P., Li M., Wen L., Zhang Y. W., Liu G. (2020). A Self-Assembled alpha-Synuclein Nanoscavenger for Parkinson's Disease. ACS Nano.

[cit4] Kim D., Yoo J. M., Hwang H., Lee J., Lee S. H., Yun S. P., Park M. J., Lee M., Choi S., Kwon S. H., Lee S., Kwon S. H., Kim S., Park Y. J., Kinoshita M., Lee Y. H., Shin S., Paik S. R., Lee S. J., Lee S., Hong B. H., Ko H. S. (2018). Graphene quantum dots prevent alpha-synucleinopathy in Parkinson's disease. Nat. Nanotechnol..

[cit5] Mahul-Mellier A. L., Burtscher J., Maharjan N., Weerens L., Croisier M., Kuttler F., Leleu M., Knott G. W., Lashuel H. A. (2020). The process of Lewy body formation, rather than simply alpha-synuclein fibrillization, is one of the major drivers of neurodegeneration. Proc. Natl. Acad. Sci. U. S. A..

[cit6] Kim C., Beilina A., Smith N., Li Y., Kim M., Kumaran R., Kaganovich A., Mamais A., Adame A., Iba M., Kwon S., Lee W. J., Shin S. J., Rissman R. A., You S., Lee S. J., Singleton A. B., Cookson M. R., Masliah E. (2020). LRRK2 mediates microglial neurotoxicity via NFATc2 in rodent models of synucleinopathies. Sci. Transl. Med..

[cit7] Ho D. H., Seol W., Son I. (2019). Upregulation of the p53-p21 pathway by G2019S LRRK2 contributes to the cellular senescence and accumulation of alpha-synuclein. Cell Cycle.

[cit8] Mead B. P., Kim N., Miller G. W., Hodges D., Mastorakos P., Klibanov A. L., Mandell J. W., Hirsh J., Suk J. S., Hanes J., Price R. J. (2017). Novel Focused Ultrasound Gene Therapy Approach Noninvasively Restores Dopaminergic Neuron Function in a Rat Parkinson's Disease Model. Nano Lett..

[cit9] Yoo J., Lee E., Kim H. Y., Youn D. H., Jung J., Kim H., Chang Y., Lee W., Shin J., Baek S., Jang W., Jun W., Kim S., Hong J., Park H. J., Lengner C. J., Moh S. H., Kwon Y., Kim J. (2017). Electromagnetized gold nanoparticles mediate direct lineage reprogramming into induced dopamine neurons in vivo for Parkinson's disease therapy. Nat. Nanotechnol..

[cit10] Yun S. P., Kam T.-I., Panicker N., Kim S., Oh Y., Park J.-S., Kwon S.-H., Park Y. J., Karuppagounder S. S., Park H., Kim S., Oh N., Kim N. A., Lee S., Brahmachari S., Mao X., Lee J. H., Kumar M., An D., Kang S.-U., Lee Y., Lee K. C., Na D. H., Kim D., Lee S. H., Roschke V. V., Liddelow S. A., Mari Z., Barres B. A., Dawson V. L., Lee S., Dawson T. M., Ko H. S. (2018). Block of A1 astrocyte conversion by microglia is neuroprotective in models of Parkinson's disease. Nat. Med..

[cit11] Liddelow S. A. (2017). Neurotoxic reactive astrocytes are induced by activated microglia. Nature.

[cit12] Jin W. N., Shi K., He W., Sun J. H., Van Kaer L., Shi F. D., Liu Q. (2021). Neuroblast senescence in the aged brain augments natural killer cell cytotoxicity leading to impaired neurogenesis and cognition. Nat. Neurosci..

[cit13] Baar M. P., Brandt R. M. C., Putavet D. A., Klein J. D. D., Derks K. W. J., Bourgeois B. R. M., Stryeck S., Rijksen Y., van Willigenburg H., Feijtel D. A., van der Pluijm I., Essers J., van Cappellen W. A., van I. W. F., Houtsmuller A. B., Pothof J., de Bruin R. W. F., Madl T., Hoeijmakers J. H. J., Campisi J., de Keizer P. L. J. (2017). Targeted Apoptosis of Senescent Cells Restores Tissue Homeostasis in Response to Chemotoxicity and Aging. Cell.

[cit14] Turnquist C., Horikawa I., Foran E., Major E. O., Vojtesek B., Lane D. P., Lu X., Harris B. T., Harris C. C. (2016). p53 isoforms regulate astrocyte-mediated neuroprotection and neurodegeneration. Cell Death Differ..

[cit15] Hu Y. L., Fryatt G. L., Ghorbani M., Obst J., Menassa D. A., Martin-Estebane M., Muntslag T. A. O., Olmos-Alonso A., Guerrero-Carrasco M., Thomas D., Cragg M. S., Gomez-Nicola D. (2021). Replicative senescence dictates the emergence of disease-associated microglia and contributes to A beta pathology. Cell Rep..

[cit16] Cai Y., Zhou H., Zhu Y., Sun Q., Ji Y., Xue A., Wang Y., Chen W., Yu X., Wang L., Chen H., Li C., Luo T., Deng H. (2020). Elimination of senescent cells by beta-galactosidase-targeted prodrug attenuates inflammation and restores physical function in aged mice. Cell Res..

[cit17] Johmura Y., Yamanaka T., Omori S., Wang T. W., Sugiura Y., Matsumoto M., Suzuki N., Kumamoto S., Yamaguchi K., Hatakeyama S., Takami T., Yamaguchi R., Shimizu E., Ikeda K., Okahashi N., Mikawa R., Suematsu M., Arita M., Sugimoto M., Nakayama K. I., Furukawa Y., Imoto S., Nakanishi M. (2021). Senolysis by glutaminolysis inhibition ameliorates various age-associated disorders. Science.

[cit18] Ungerleider K., Beck J., Lissa D., Turnquist C., Horikawa I., Harris B. T., Harris C. C. (2021). Astrocyte senescence and SASP in neurodegeneration: tau joins the loop. Cell Cycle.

[cit19] Da Mesquita S., Louveau A., Vaccari A., Smirnov I., Cornelison R. C., Kingsmore K. M., Contarino C., Onengut-Gumuscu S., Farber E., Raper D., Viar K. E., Powell R. D., Baker W., Dabhi N., Bai R., Cao R., Hu S., Rich S. S., Munson J. M., Lopes M. B., Overall C. C., Acton S. T., Kipnis J. (2018). Functional aspects of meningeal lymphatics in ageing and Alzheimer's disease. Nature.

[cit20] Zhu L., Sun C., Ren J., Wang G., Ma R., Sun L., Yang D., Gao S., Ning K., Wang Z., Chen X., Chen S., Zhu H., Gao Z., Xu J. (2019). Stress-induced precocious aging in PD-patient iPSC-derived NSCs may underlie the pathophysiology of Parkinson's disease. Cell Death Discovery.

[cit21] Chinta S. J., Woods G., Demaria M., Rane A., Zou Y., McQuade A., Rajagopalan S., Limbad C., Madden D. T., Campisi J., Andersen J. K. (2018). Cellular Senescence Is Induced by the Environmental Neurotoxin Paraquat and Contributes to Neuropathology Linked to Parkinson's Disease. Cell Rep..

[cit22] Shaerzadeh F., Phan L., Miller D., Dacquel M., Hachmeister W., Hansen C., Bechtle A., Tu D., Martcheva M., Foster T. C., Kumar A., Streit W. J., Khoshbouei H. (2020). Microglia senescence occurs in both substantia nigra and ventral tegmental area. Glia.

[cit23] Zhang P., Kishimoto Y., Grammatikakis I., Gottimukkala K., Cutler R. G., Zhang S., Abdelmohsen K., Bohr V. A., Misra Sen J., Gorospe M., Mattson M. P. (2019). Senolytic therapy alleviates Abeta-associated oligodendrocyte progenitor cell senescence and cognitive deficits in an Alzheimer's disease model. Nat. Neurosci..

[cit24] Bussian T. J., Aziz A., Meyer C. F., Swenson B. L., van Deursen J. M., Baker D. J. (2018). Clearance of senescent glial cells prevents tau-dependent pathology and cognitive decline. Nature.

[cit25] Riessland M., Kolisnyk B., Kim T. W., Cheng J., Ni J., Pearson J. A., Park E. J., Dam K., Acehan D., Ramos-Espiritu L. S., Wang W., Zhang J., Shim J. W., Ciceri G., Brichta L., Studer L., Greengard P. (2019). Loss of SATB1 Induces p21-Dependent Cellular Senescence in Post-mitotic Dopaminergic Neurons. Cell Stem Cell.

[cit26] Qu A., Sun M., Kim J. Y., Xu L., Hao C., Ma W., Wu X., Liu X., Kuang H., Kotov N. A., Xu C. (2021). Stimulation of neural stem cell differentiation by circularly polarized light transduced by chiral nanoassemblies. Nat. Biomed. Eng..

[cit27] Hou K., Zhao J., Wang H., Li B., Li K., Shi X., Wan K., Ai J., Lv J., Wang D., Huang Q., Wang H., Cao Q., Liu S., Tang Z. (2020). Chiral gold nanoparticles enantioselectively rescue memory deficits in a mouse model of Alzheimer's disease. Nat. Commun..

[cit28] Lv J., Gao X., Han B., Zhu Y., Hou K., Tang Z. (2022). Self-assembled inorganic chiral superstructures. Nat. Rev. Chem..

[cit29] Tang H., Li Q., Yan W., Jiang X. (2021). Reversing the Chirality of Surface Ligands Can Improve the Biosafety and Pharmacokinetics of Cationic Gold Nanoclusters. Angew. Chem., Int. Ed. Engl..

[cit30] Wang W., Zhao J., Hao C., Hu S., Chen C., Cao Y., Xu Z., Guo J., Xu L., Sun M., Xu C., Kuang H. (2022). The development of chiral nanoparticles to target NK cells and CD8+ T cells for cancer immunotherapy. Adv. Mater..

[cit31] Xu L., Wang X., Wang W., Sun M., Choi W. J., Kim J.-Y., Hao C., Li S., Qu A., Lu M., Wu X., Colombari F. M., Gomes W. R., Blanco A. L., de Moura A. F., Guo X., Kuang H., Kotov N. A., Xu C. (2022). Enantiomer-dependent immunological response to chiral nanoparticles. Nature.

[cit32] Lu M., Qu A., Li S., Sun M., Xu L., Kuang H., Xu C. (2020). Mitochondria-Targeting Plasmonic Spiky Nanorods Increase the Elimination of Aging Cells in Vivo. Angew. Chem., Int. Ed. Engl..

[cit33] Si L., Maozhong S., Changlong H., Aihua Q., Xiaoling W., Liguang X., Chuanlai X., Hua K. (2020). Chiral Cux Coy S Nanoparticles under Magnetic Field and NIR Light to Eliminate Senescent Cells. Angew. Chem., Int. Ed. Engl..

[cit34] Zhu J., Wu F., Han Z., Shang Y., Liu F., Yu H., Yu L., Li N., Ding B. (2021). Strong Light-Matter Interactions in Chiral Plasmonic-Excitonic Systems Assembled on DNA Origami. Nano Lett..

[cit35] Chen G., Cao Y., Tang Y., Yang X., Liu Y., Huang D., Zhang Y., Li C., Wang Q. (2020). Advanced Near-Infrared Light for Monitoring and Modulating the Spatiotemporal Dynamics of Cell Functions in Living Systems. Adv. Sci..

[cit36] Liu H., Han Y., Wang T., Zhang H., Xu Q., Yuan J., Li Z. (2020). Targeting Microglia for Therapy of Parkinson's Disease by Using Biomimetic Ultrasmall Nanoparticles. J. Am. Chem. Soc..

[cit37] Wang Y., Xie Y., Luo J., Guo M., Hu X., Chen X., Chen Z., Lu X., Mao L., Zhang K., Wei L., Ma Y., Wang R., Zhou J., He C., Zhang Y., Zhang Y., Chen S., Shen L., Chen Y., Qiu N., Liu Y., Cui Y., Liao G., Liu Y., Chen C. (2021). Engineering
a self-navigated MnARK nanovaccine for inducing potent protective immunity against novel coronavirus. Nano Today.

[cit38] Wu J., Cui X., Ke P. C., Mortimer M., Wang X., Bao L., Chen C. (2021). Nanomaterials as novel agents for amelioration of Parkinson’s disease. Nano Today.

[cit39] Huang D., Cao Y., Yang X., Liu Y., Zhang Y., Li C., Chen G., Wang Q. (2021). A Nanoformulation-Mediated Multifunctional Stem Cell Therapy with Improved Beta-Amyloid Clearance and Neural Regeneration for Alzheimer's Disease. Adv. Mater..

[cit40] Qu A., Wu X., Li S., Sun M., Xu L., Kuang H., Xu C. (2020). An NIR-Responsive DNA-Mediated Nanotetrahedron Enhances the Clearance of Senescent Cells. Adv. Mater..

[cit41] Anderson S. R., Roberts J. M., Zhang J. M., Steele M. R., Romero C. O., Bosco A., Vetter M. L. (2019). Developmental Apoptosis Promotes a Disease-Related Gene Signature and Independence from CSF1R Signaling in Retinal Microglia. Cell Rep..

[cit42] Ray P., Guha D., Chakraborty J., Banerjee S., Adhikary A., Chakraborty S., Das T., Sa G. (2016). Crocetin exploits p53-induced death domain (PIDD) and FAS-associated death domain (FADD) proteins to induce apoptosis in colorectal cancer. Sci. Rep..

[cit43] Cruz A. C., Ramaswamy M., Ouyang C., Klebanoff C. A., Sengupta P., Yamamoto T. N., Meylan F., Thomas S. K., Richoz N., Eil R., Price S., Casellas R., Rao V. K., Lippincott-Schwartz J., Restifo N. P., Siegel R. M. (2016). Fas/CD95 prevents autoimmunity independently of lipid raft localization and efficient apoptosis induction. Nat. Commun..

[cit44] Xi W., Saw T. B., Delacour D., Lim C. T., Ladoux B. (2018). Material approaches to active tissue mechanics. Nat. Rev. Mater..

[cit45] Asbury C. L., Gestaut D. R., Powers A. F., Franck A. D., Davis T. N. (2006). The Dam1 kinetochore complex harnesses microtubule dynamics to produce force and movement. Proc. Natl. Acad. Sci. U. S. A..

[cit46] Chen S., Weitemier A. Z., Zeng X., He L., Wang X., Tao Y., Huang A. J. Y., Hashimotodani Y., Kano M., Iwasaki H., Parajuli L. K., Okabe S., Teh D. B. L., All A. H., Tsutsui-Kimura I., Tanaka K. F., Liu X., McHugh T. J. (2018). Near-infrared deep brain stimulation via upconversion nanoparticle-mediated optogenetics. Science.

[cit47] Sharma S., Carmona A., Skowronek A., Yu F., Collins M. O., Naik S., Murzeau C. M., Tseng P. L., Erdmann K. S. (2019). Apoptotic signalling targets the post-endocytic sorting machinery of the death receptor Fas/CD95. Nat. Commun..

