# Supplementary material for: Photoinduced elimination of senescent microglia cells in vivo by chiral gold nanoparticles
Source: Chem Sci. 2022 May 16;13(22):6642–54. doi: 10.1039/d2sc01662a (PMC9172567; doi:10.1039/d2sc01662a)
Supplement: SC-013-D2SC01662A-s001 [file SC-013-D2SC01662A-s001.pdf]

Electronic Supplementary Material (ESI) for Chemical Science.
This journal is © The Royal Society of Chemistry 2022

Supporting information

Materials and instruments

All chemicals and reagents were used as received. Hydrogen tetrachloroaurate trihydrate ($HAuCl_4 \cdot 3H_2O$), cetyltrimethylammonium bromide (CTAB), hexadecyl trimethyl ammonium chloride (CTAC), sodium borohydride ($NaBH_4$), sodium hydroxide (NaOH) and ascorbic acid were purchased from Sigma-Aldrich (St. Louis, MO, USA). 1-ethyl-3-(3-dimethylaminopropyl) carbodiimide (EDC), and N-hydroxysuccinimide (NHS) and potassium iodide (KI) were purchased from Aladdin Chemistry Co., Ltd. (Shanghai, China). Cysteine-phenylalanine (CF) dipeptide was synthesized by Sangon Biotechnology, Shanghai. Milli-Q water (> 18 MΩ cm) was used in all experiments. All ELISA kits were purchased from Jiangsu Jingmei Biological Technology Co.,Ltd.

The synthesis of chiral gold NPs

Using a typical synthetic process, we synthesized the gold nanoprisms as seeds[1]. The nanoprisms were synthesized by following a set protocol. First, 78 μM KI and 16.7 mM CTAC were mixed in 10 mL water. Then, sodium tetrachloroaurate solution was prepared by mixing 10 mM $HAuCl_4$ and 10 mM NaOH. Next, the 100.4 μL of the prepared sodium tetrachloroaurate solution was added into the reaction solution. Finally, 10 μL of 0.1M NaOH was rapidly injected and stirred vigorously for 30 s. The nanoprisms were then obtained by centrifugation.

Next, a growth solution was formed by adding 0.08 mL of 100 mM CTAB to 3.95 mL of deionized water. Then, 0.2 mL of 10 mM $HAuCl_4$ was added to form an $AuBr^{4-}$ complex. After incubation for 5 min, 0.475 mL of 50 mM VC and 5μL of 4mM CF dipeptide were injected rapidly to the growth solution. Finally, 100μL of seeds was added with vigorously stringing. Polarized light (light wavelength: 594 nm; intensity: 84 mW/cm^2) was applied during the growth process. The reaction solution was immediately illuminated with various types of polarized light for 30 min immediately after the injection of seeds. Right circularly polarized light (RCP) was applied to form D-P$^-$ NPs and left circularly polarized light (LCP) was applied to form L-P$^+$ NP. As for the synthesis of DL-NP, DL-CF and 594 nm laser were used following the same steps. Finally, the chiral NPs were modified with 4 mM mPEG-SH (MW: 2000) for 4 h for further biological application.

Preparation of Au@anti-B2MG and anti-DCR2

We prepared different concentration of anti-DCR2 (0, 0.4, 0.6, 0.8, and 1.0 nM) and anti-B2MG in 200 μL DMF and mixed these with 1.89 mg of EDC and 0.57 mg of NHS. The solution was stirred for 4h at 37 °C in the dark. Then the mixture was added dropwise into the NPs and the solution was stirred for 6 h at 37 °C in the dark. Finally, unconjugated antibodies were removed by centrifugation at 1700×g for 3 min. The successful conjugation of NPs and antibodies was characterized by CD and the UV spectrum.

Cell lines and incubation conditions

All cells were cultured at 37 °C in a humidified atmosphere of 5% CO_2 and 95% air. MA-C and MN9D cells were grown in DMEM medium with 1.0 mg/mL d-glucose supplemented with 10% FBS and 1% penicillin streptomycin. BV-2 cells were grown in DMEM medium with 4.5 mg/mL d-glucose supplemented with 10% FBS and 1% penicillin streptomycin.

Cellular survival assays for nanoparticles

BV-2, MA-C and MN9D cells were placed in 96-well plates and incubated for 24 h. Then, new culture medium, containing different concentrations of nanomaterials, was added to replace the old medium. After 24 h of incubation, cell viability was assessed using the standard Cell Counting Kit-8 (CCK-8) in accordance with the manufacturer's instructions (Beyotime).

Clearance of senescent cell *in vitro*

The clearance of senescent cells *in vitro* was determined by the fluorescence intensity of caspases-3, a known marker of apoptosis. Generally, cells were fixed with 4% paraformaldehyde for 15 min, permeabilized with 0.1% Triton X-100 for 2 min, and then blocked with 1% bovine serum albumin (BSA) for 2 h at 20 °C. Then, the cells were washed three times in PBS before being incubated with anti-caspase-3 for another 2 h at 26 °C. Subsequently, the cells were washed with PBS and then incubated with Alex488-conjugated (goat-anti-rabbit) secondary antibody (1:500) for 2 h at 26 °C.

After washing, the cells were imaged using a Leica LSM880 confocal fluorescence microscope.

Western blot

BV-2 cells (1.0 × 10^6) of different treatment were collected. Total protein was extracted by RIPA (Sangon Biotech Co., Ltd.), in accordance with the manufacturer's instructions. The primary antibodies used in western blot were anti-caspase-3 (1:1000), anti-GADPH (1:1000), anti-caspase-8 (1:500), anti-Fas and a horseradish-peroxidase-conjugated secondary antibody (1:500). GAPDH was set as control.

Flow cytometry assays

Flow cytometry was used to detect the proportion of apoptotic cells. BV-2, MA-C and MN9D cells (1.0×106) undergoing different treatments were collected and washed with PBS. The detection of apoptosis in MN9D cells was carried out with an Annexin V-FITC Apoptosis detection kit (Beyotime), in accordance with the manufacturer's instructions.

Transcriptomic genes analysis

RNA was extracted from BV-2 cells with Total RNA Extractor (Sangon Biotechnology, Shanghai) in accordance with the manufacturer's protocol. In total, 10^6 cells were collected and washed three times in PBS. Then, 1mL of Total RNA Extractor was added immediately. Each sample was stored at – 80 °C for further treatment.

Rotarod test

All animal experiments were approved by the Committee on Animal Welfare of Jiangnan University. The rotarod test was carried out to determine the motor ability of PD mice undergoing different treatments. For the rotarod test, mice were placed on a rotarod cylinder and the speed was slowly increased speed from 4 to 40 rpm over a 5 min period. We then measured the amount of time each animal remained on the rotarod at least three times after training for 5 days.

Morris water maze test

The Morris water maze test was used to investigate the memory of PD mice undergoing different treatments. The training phase was carried out four times per day for 5 continuous days, in which we tested the ability of the animals to find the position of the platform. We recorded the latency for escape to the platform was recorded. After training for 5 days, the spatial memory of the mice was characterized by the proportion of time spent in the target, the time spent in the target quadrant, and the number of platform crossings in a probe test.

Running wheel test

The running wheel test was used to monitor the improvement of the movement disorder of PD mice undergoing different treatments. The training phase was carried out for 5 continuous days to allow the mice to become familiar with the running wheel. Then,

mice were given 60-min periods of wheel-running exercise. For each mouse, we recoded the maximum speed and calculative distance in 60 min.

Immunofluorescence and immunohistochemistry staining

The brains of each mice were stored in 10% formaldehyde and then sectioned into thin slices. Each piece was fixed with 4% paraformaldehyde for 15 min, permeabilized with 0.1% Triton X-100 for 10 min and blocked overnight with 1% BSA at 4°C. Then, brain sections were incubated with IBA-1 and TH antibodies (1:500) at 20°C. Subsequently, sections were washed with PBS and then incubated with Alex488-conjugated (goat-anti-rabbit) and Alex555-conjugated (goat-anti-mouse) secondary antibodies (1:500) for 2 h at room temperature. After washing with PBS, the cells were imaged using a Leica LSM880 confocal fluorescence microscope. The same antibodies were used for IHC.

Histological examination

An H&E staining kit was used to investigate the biocompatibility of gold nanoparticles in major organs

Statistical analyzes

All data are presented as mean ± standard deviation. For each experimental series, at least five replicates (n = 5) were performed unless otherwise stated. For many experimental series at least five independent experiments were carried out; n values

given in the figure legends indicate the number of independent experiments performed or the number of mice used. Group differences were considered statistically significant when $p < 0.05$ (*$p < 0.05$, **$p < 0.01$, ***$p < 0.001$). Analysis of variance test was used for multicomponent comparison.

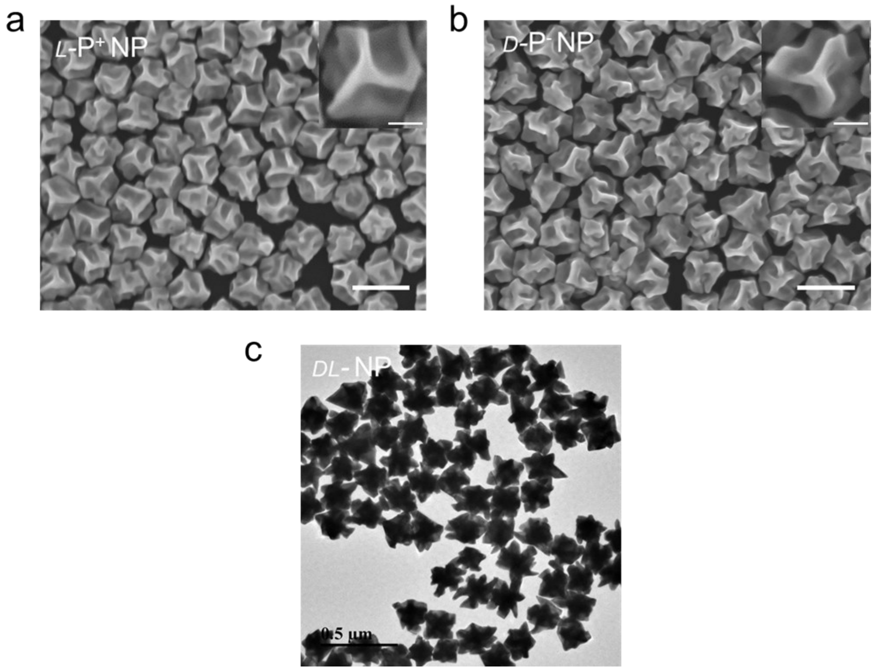

Figure S1. The SEM image of (**a**) L-P$^+$ NP and (**b**) D-P$^-$ NP. Scale bars, 200 nm. Scale bars in insert images, 50 nm. The TEM image of (**c**) DL-NP. Scale bars, 500 nm.

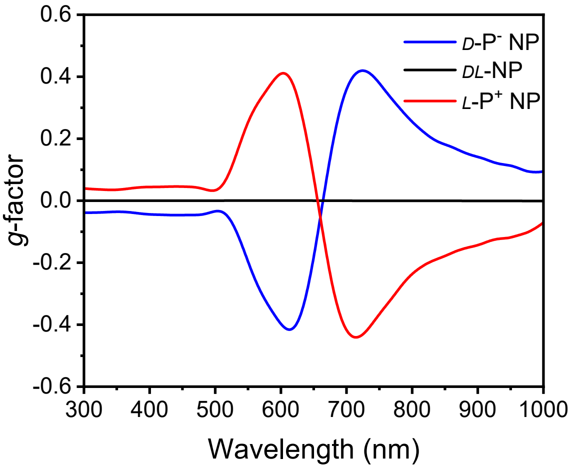

Figure S2. The *g*-factor spectrums of chiral NPs.

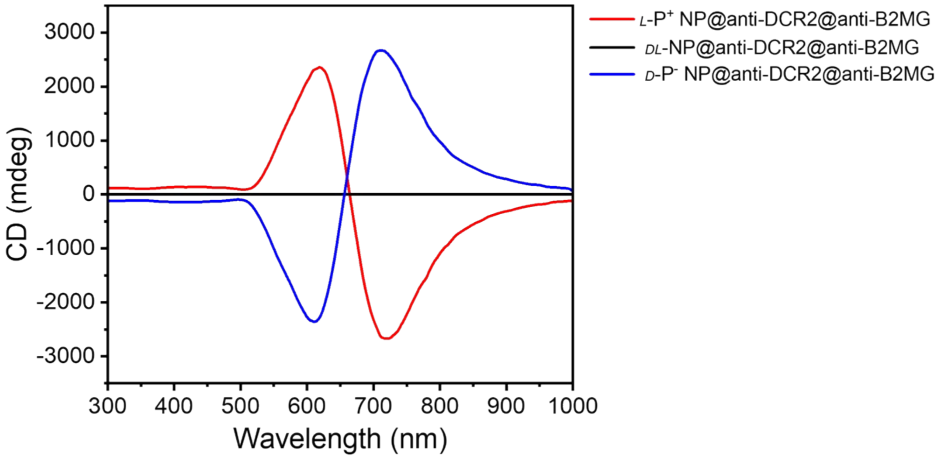

Figure S3. The CD spectrum of *L*-P$^+$ NP@anti-DCR2@anti-B2MG, *DL*-NP@anti-DCR2@anti-B2MG and *D*-P$^-$ NP@anti-DCR2@anti-B2MG in 300-1000 nm range.

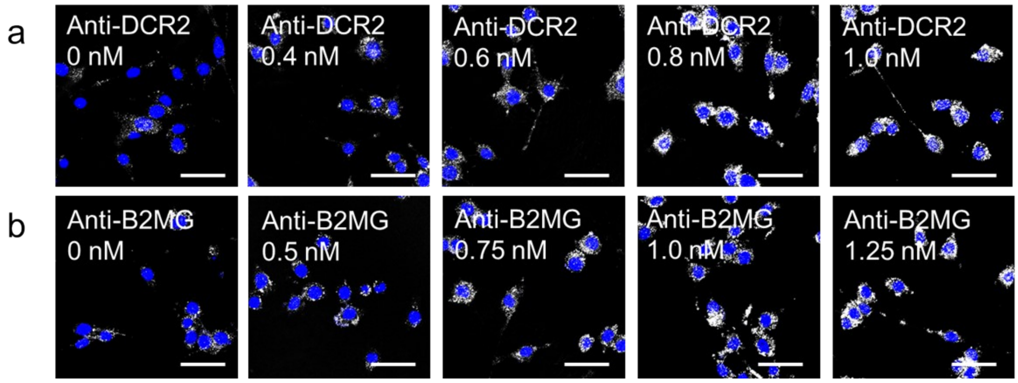

Figure S4. (**a**) The TPL images of senescent BV-2 cells incubated with the *L*-P$^+$ NP conjected with different concentration of anti-DCR2 and 1 nM anti-B2MG for 6 h. (**b**) The TPL images of senescent BV-2 cells incubated with the *L*-P$^+$ NP conjected with different concentration of anti-B2MG and 0.8nM anti-DCR2 for 6 h. Scale bars, 30 μm.

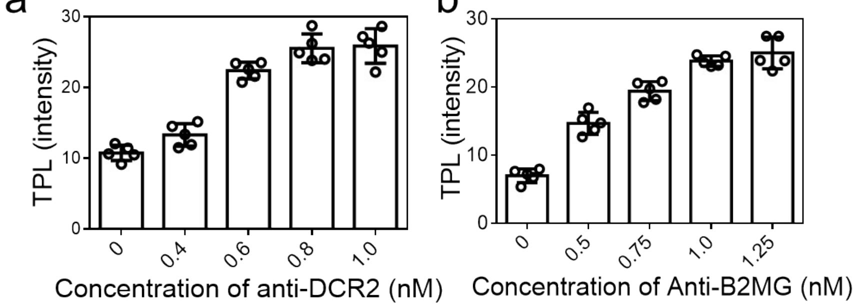

Figure S5. The TPL intensity of NPs (**a**) from **Figure S4a** and (**b**) **Figure S4b**. Data are presented as mean ± s. d. (*n* = 5).

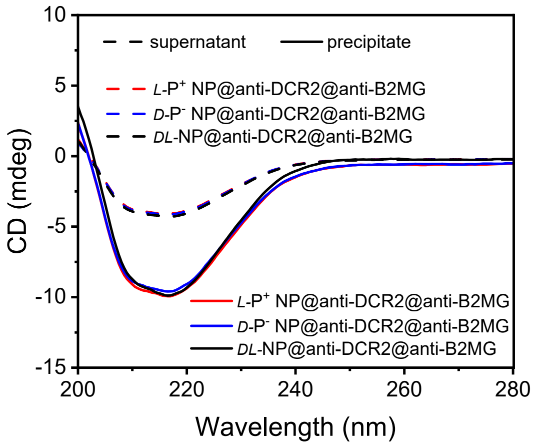

Figure S6. The CD spectrum of supernatant and precipitate of L-P$^+$ NP, DL-NP and D-P$^-$ NP when incubated with anti-DCR2 and anti-B2MG after separation.

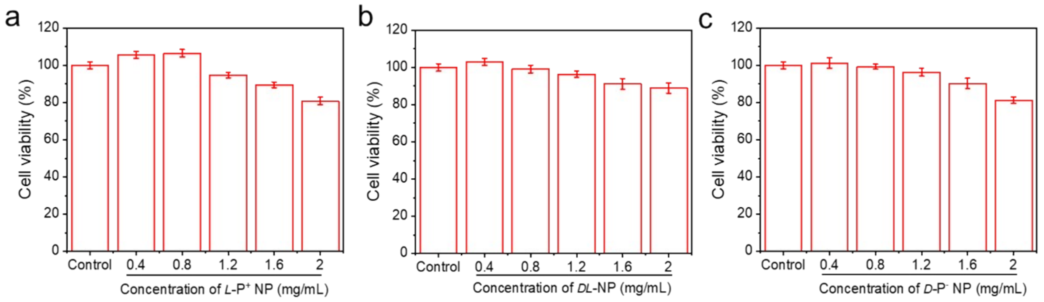

Figure S7. Viability of senescent BV-2 cells incubated with different concentration of (**a**) L-P$^+$ NP, (**b**) DL-NP, (**c**) D-P$^-$ NP. Data are presented as mean ± s. d. (*n* = 5).

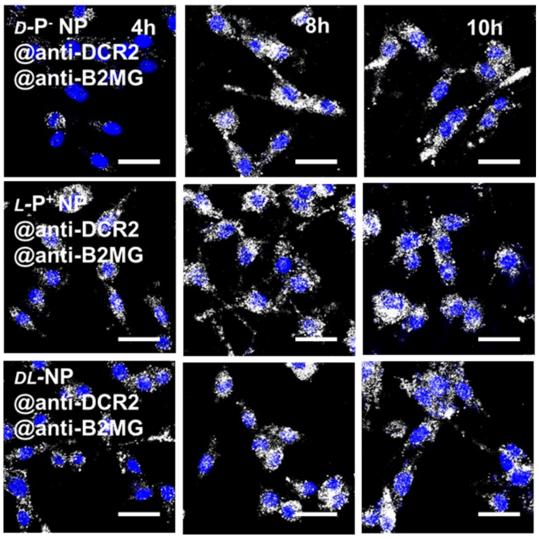

Figure S8. TPL images of senescent BV-2 cells incubated with chiral NPs coated with antibodies for different time respectively. Scale bars, 30 μm.

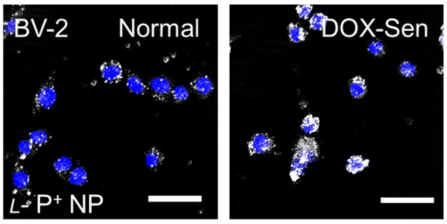

Figure S9. TPL images of *L*-P$^+$ NP coated with antibodies in normal and senescent BV-2 cells with incubation for 3h. Scale bars, 30 μm.

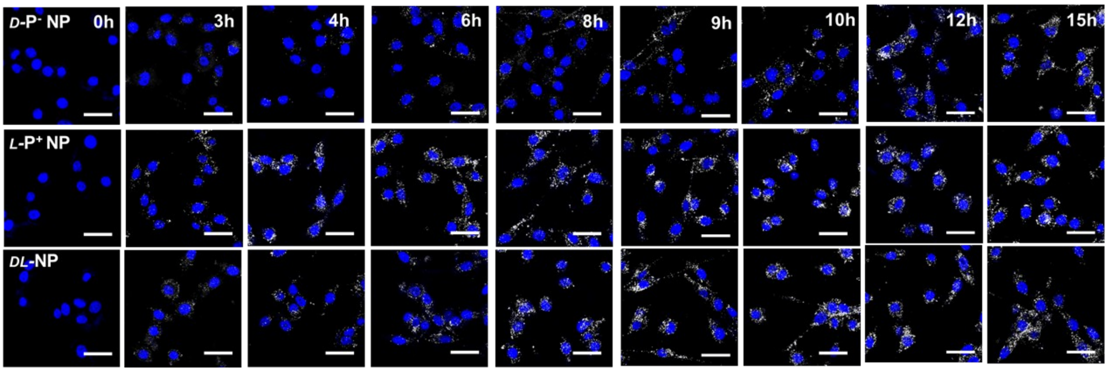

Figure S10. TPL images of senescent BV-2 cells incubated with chiral NPs without antibody coating for different time respectively. Scale bars, 30 μm.

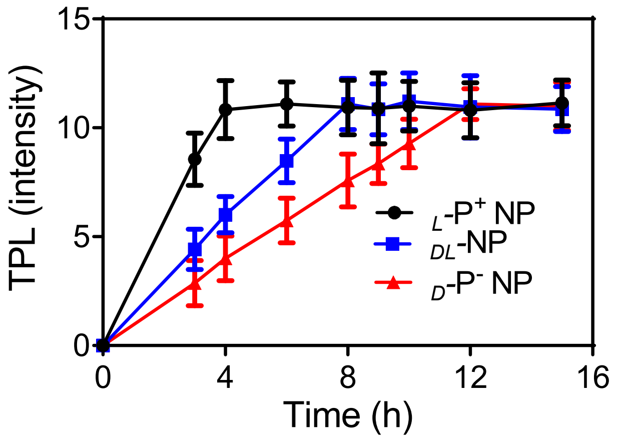

Figure S11. The statistic intensity from the TPL images in **Figure S10**.

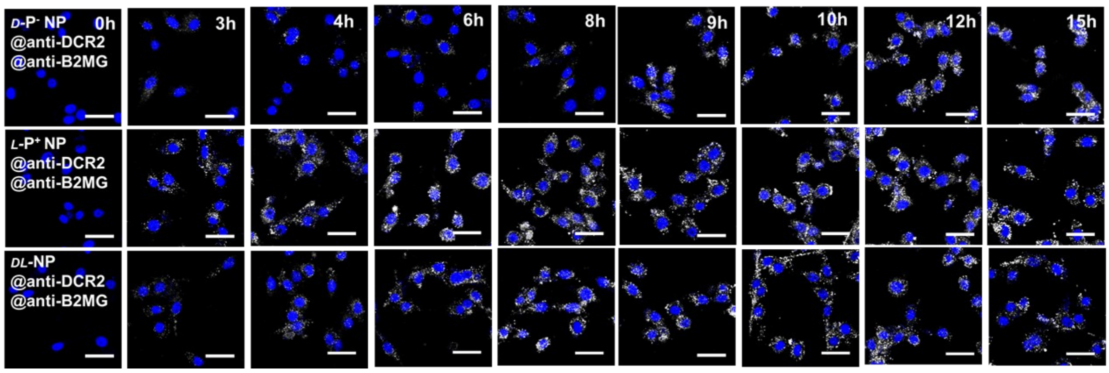

Figure S12. TPL images of normal BV-2 cells incubated with chiral NPs coated with antibodies for different time respectively. Scale bars, 30 μm.

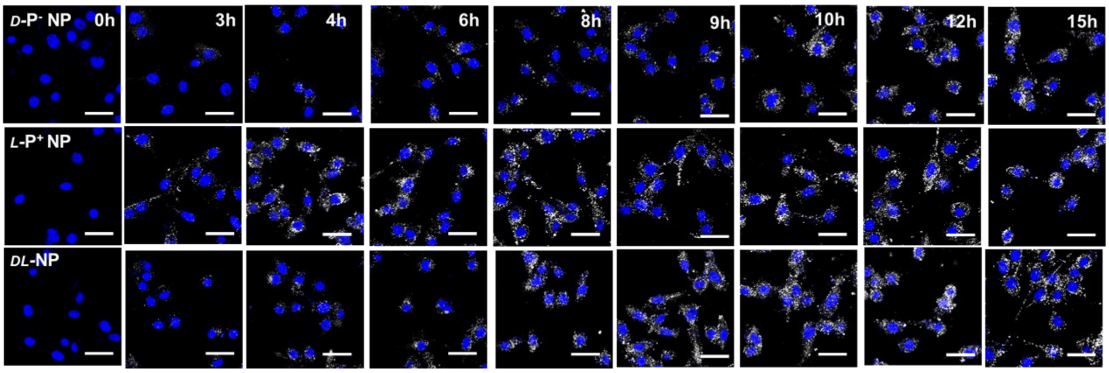

Figure S13. TPL images of normal BV-2 cells incubated with chiral NPs for different time respectively. Scale bars, 30 μm.

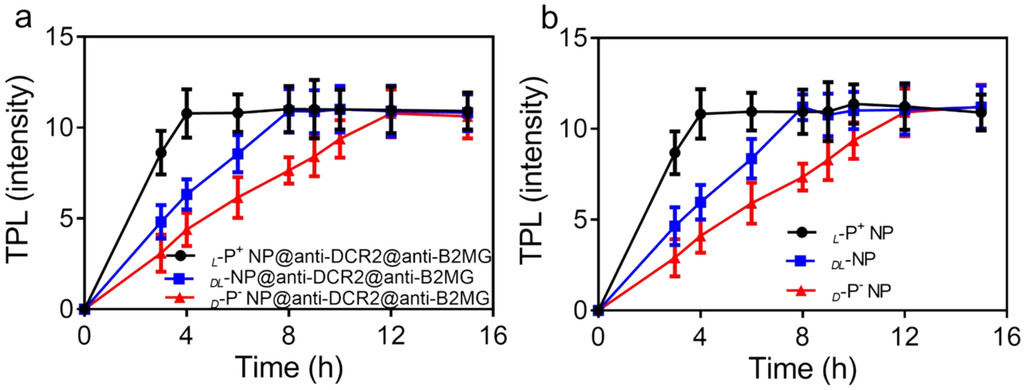

Figure S14. The statistic intensity from the TPL images in (**a**) **Figure S12** and **(b) Figure S13.**

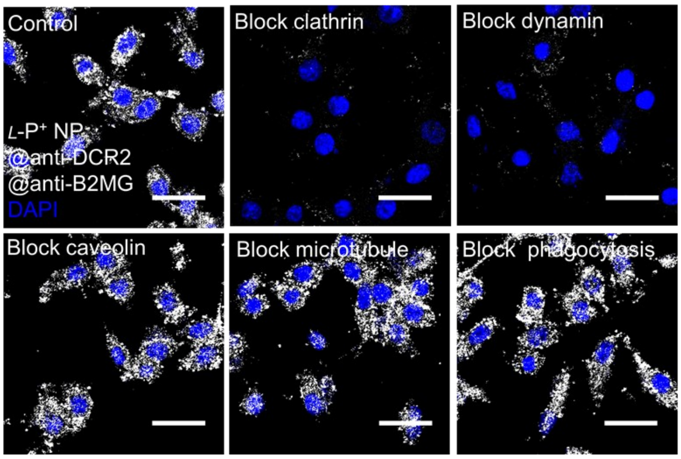

Figure S15. TPL images of *L*-P$^+$ NP @anti-DCR2@anti-B2MG in senescent BV-2 cells after incubation in PBS (control), cytochalasin D (phagocytosis inhibitor), nocodazole (microtubule inhibitor), dynasore (dynamin inhibitor), chlorpromazine (clathrin inhibitor). Scale bars, 30 μm.

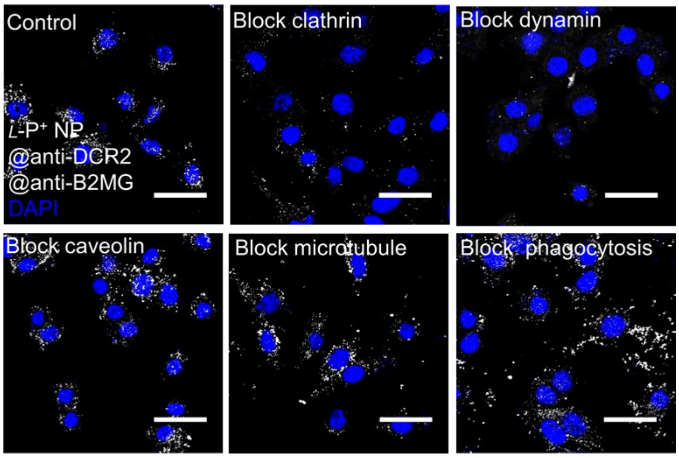

Figure S16. TPL images of *L*-P$^+$ NP @anti-DCR2@anti-B2MG in normal BV-2 cells after incubation in PBS (control), cytochalasin D (phagocytosis inhibitor), nocodazole (microtubule inhibitor), dynasore (dynamin inhibitor), chlorpromazine (clathrin inhibitor). Scale bars, 30 μm.

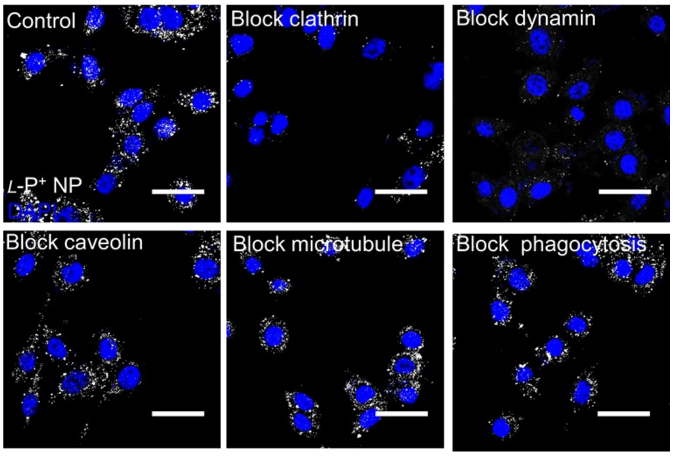

Figure S17. TPL images of *L*-P$^+$ NP in senescent BV-2 cells after incubation in PBS (control), cytochalasin D (phagocytosis inhibitor), nocodazole (microtubule inhibitor), dynasore (dynamin inhibitor), chlorpromazine (clathrin inhibitor). Scale bars, 30 μm.

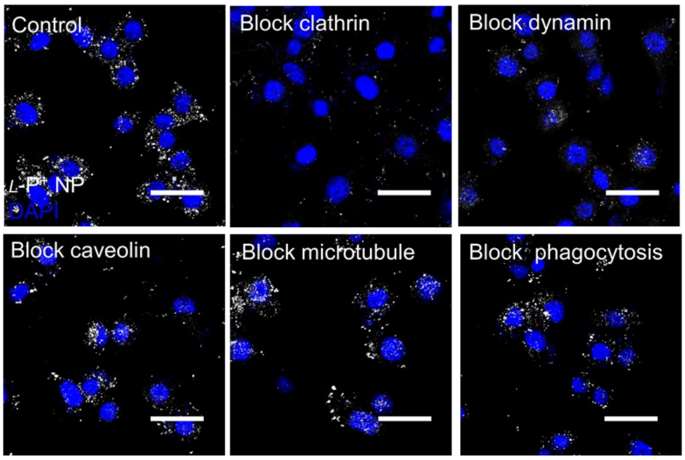

Figure S18. TPL images of *L*-P$^+$ NP in normal BV-2 cells after incubation in PBS (control), cytochalasin D (phagocytosis inhibitor), nocodazole (microtubule inhibitor), dynasore (dynamin inhibitor), chlorpromazine (clathrin inhibitor). Scale bars, 30 μm.

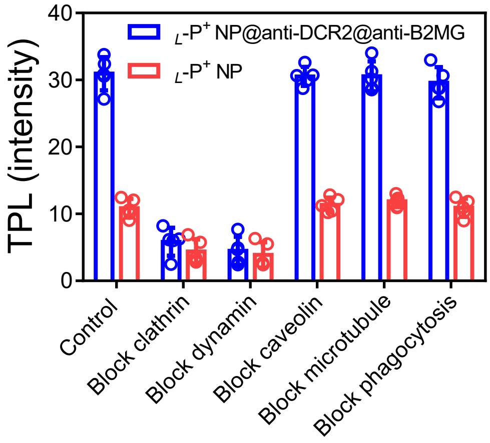

Figure S19. The statistic intensity from the TPL images in **Figure S15 and S17.**

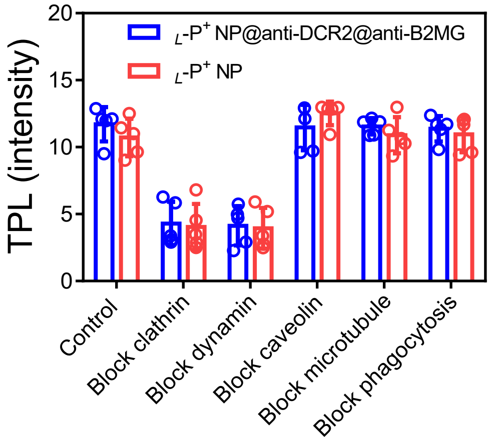

Figure S20. The statistic intensity from the TPL images in **Figure S16 and S18.**

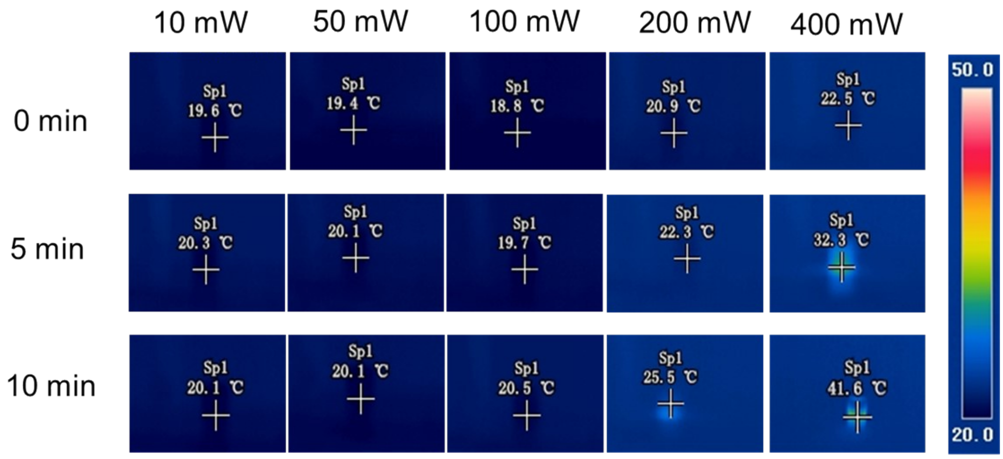

Figure S21. Thermal imaging of *L*-P$^+$ NP under different irradiation in living cells (10, 50, 100, 200 and 400 mW/cm^2) of different time (0, 5, 10 min). The 808 nm laser of 400 mW/cm^2 5 min was selected in following experiments.

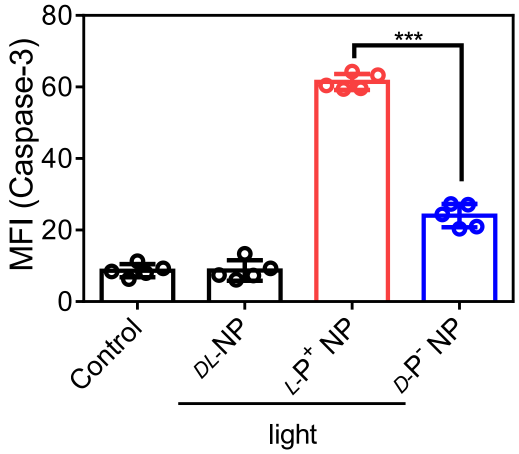

Figure S22. The statistic of caspase-3 for the result in **Figure 3a** of senescent BV-2 cells. Data are presented as mean ± s. d. (n = 5). ***p<0.001.

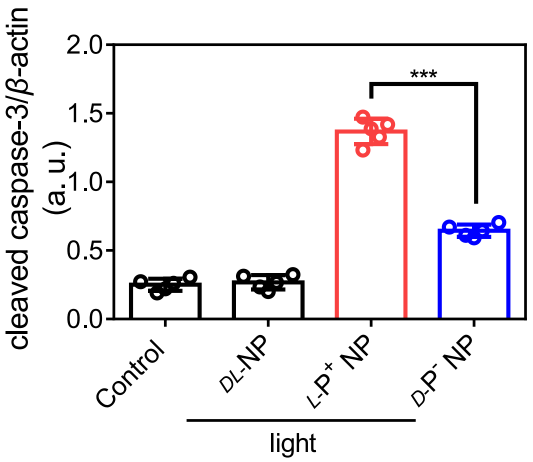

Figure S23. Results of western blot for cleaved caspase-3 in senescent BV-2 cells with different treatments in **Figure 3b**. Data are presented as mean ± s. d. (n = 5). ***p<0.001.

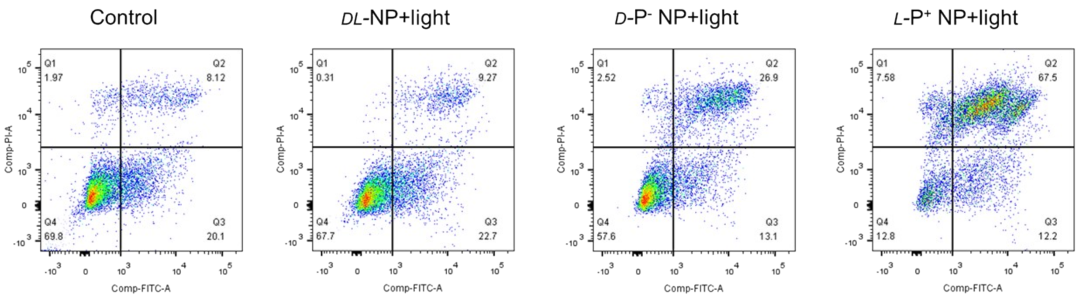

Figure S24. Flow cytometry scatter plot representing apoptosis assay based on Annexin V-FITC/PI staining senescent BV-2 cells with different treatments. Senescent BV-2 cells without any treatment were set as control.

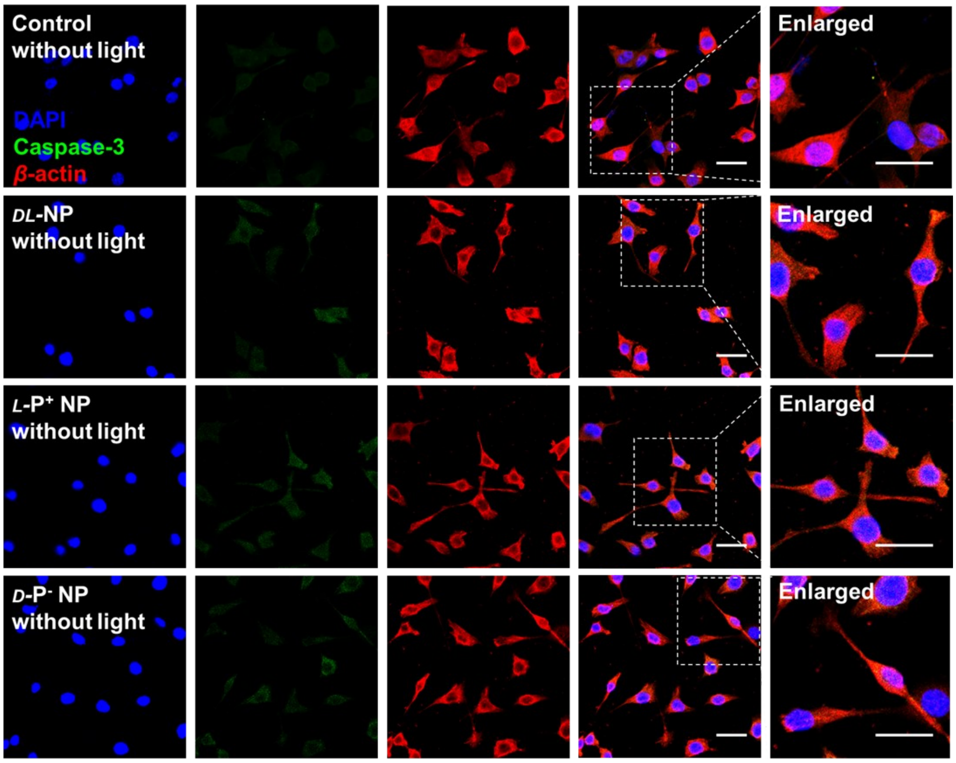

Figure S25. Confocal images of senescent BV-2 cells with NPs coated with antibodies without light. Senescent BV-2 cells without any treatment were set as control. Red, β-actin; green, caspase-3; blue, DAPI for nuclei. Scale bars, 40 μm.

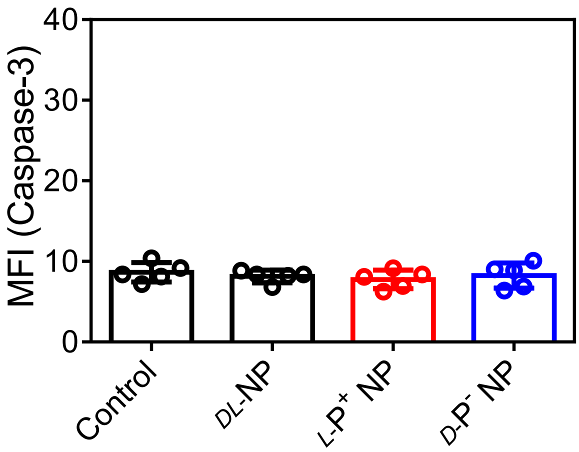

Figure S26. MFI of caspase-3 for the result in **Figure S25** of senescent BV-2 cells. Data are presented as mean ± s. d. (n = 5).

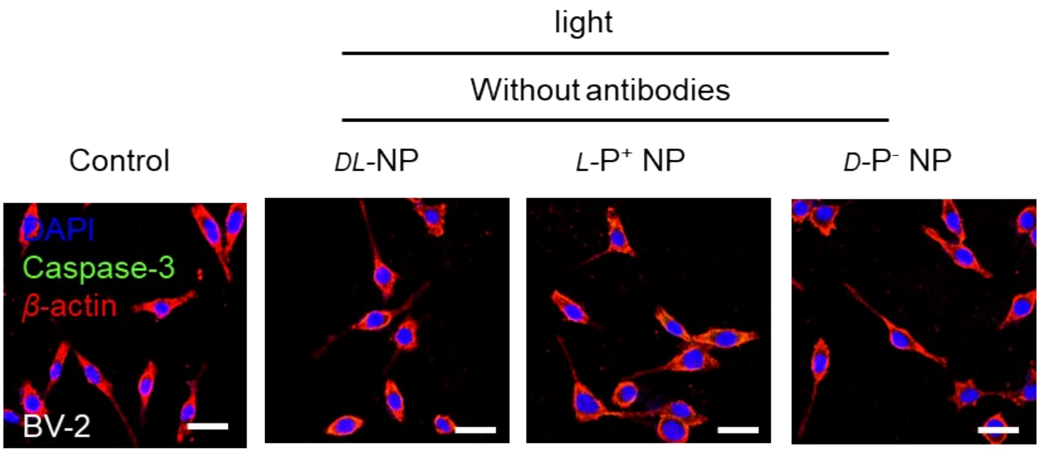

Figure S27. Confocal images of senescent BV-2 cells incubated with chiral NPs without anti-DCR2 and anti-B2MG coated under light. Senescent cells without any treatment were set as control. Red, β-actin; green, caspase-3; blue, DAPI for nuclei. Scale bars, 40 μm.

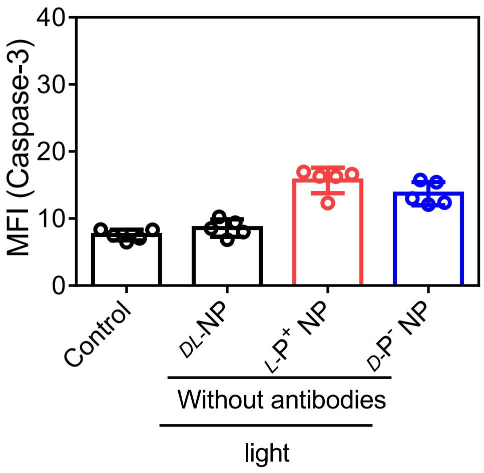

Figure S28. MFI of caspase-3 for the results in **Figure S27** of senescent BV-2 cells. Data are presented as mean ± s. d. (n = 5).

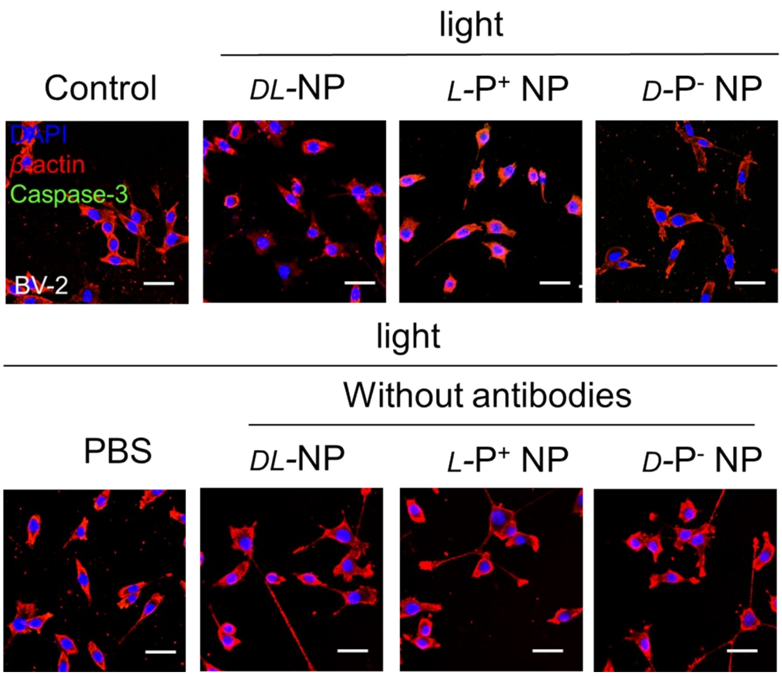

Figure S29. Confocal images of normal BV-2 cells incubated with different groups under light. Red, β-actin; green, caspase-3; blue, DAPI for nuclei. Scale bars, 40 µm.

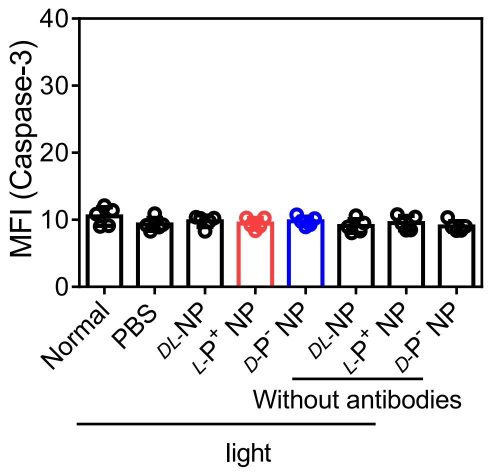

Figure S30. MFI of caspase-3 for the result in **Figure S29** of different BV-2 cells. Data are presented as mean ± s. d. (n = 5).

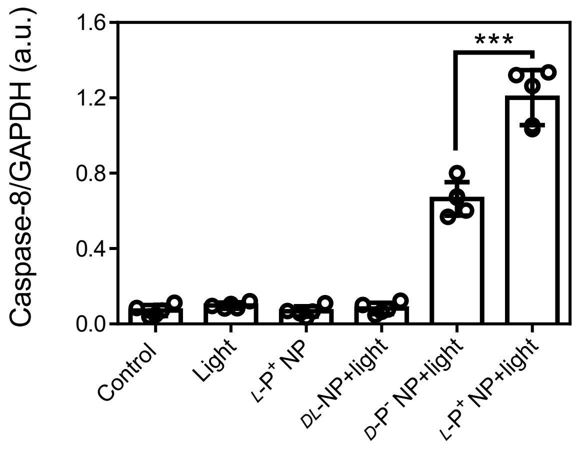

Figure S31. Results of western blot for cleaved caspase-8 in senescent BV-2 cells with different treatments in **Figure 4c**. Data are presented as mean ± s. d. (n = 5). ***$p<0.001$.

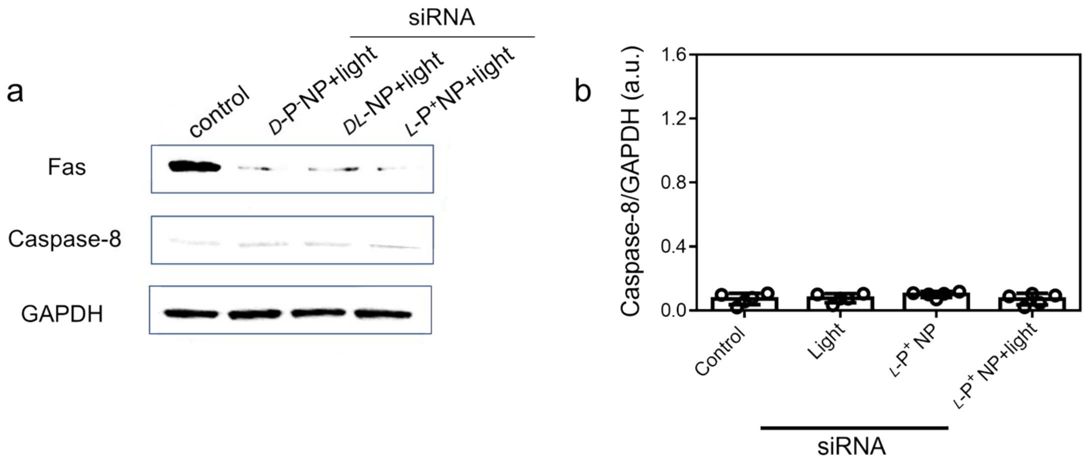

Figure S32. (**a**) Western blot analysis for Fas and cleaved caspase-8 in senescent BV-2 cells with different treatments, GAPDH was set as control. (**b**) Results of western blot for cleaved caspase-8 in senescent BV-2 cells with different treatments in **a**.

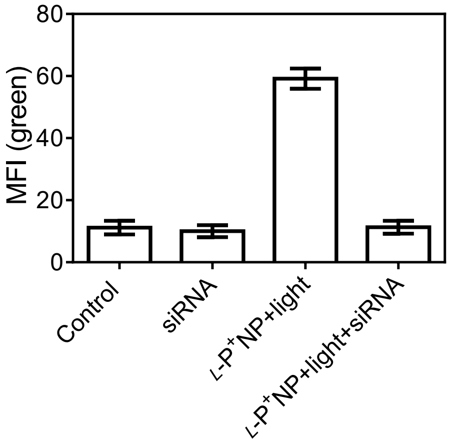

Figure S33. MFI of caspase-3 for the result in **Figure 4e** of senescent BV-2 cells with different treatments. Data are presented as mean ± s. d. (n = 5).

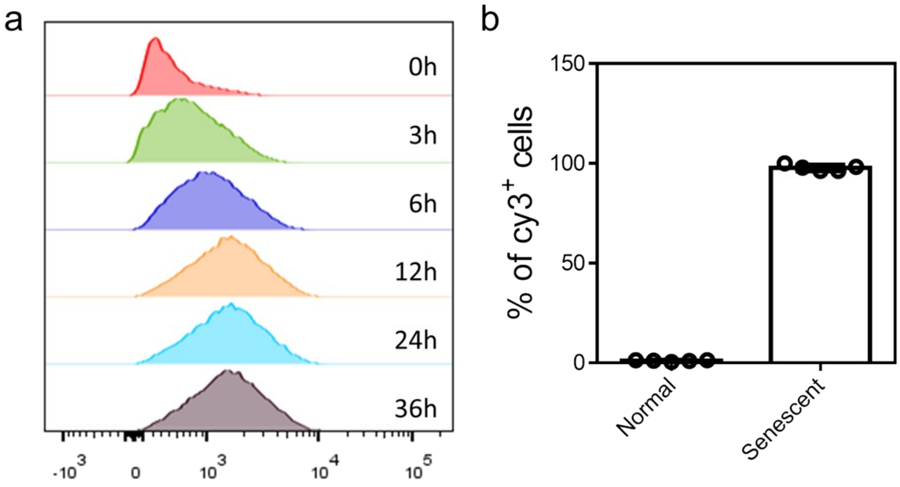

Figure S34. (a) The amount of *L*-P^{+} NP@anti-DCR2@anti-B2MG in the brain of PD mic after injection for different time by flow cytometry. (**b**) Detection of Cy3^{+} normal and senescent cells in the brain of PD mice after injection for 12h by flow cytometry.

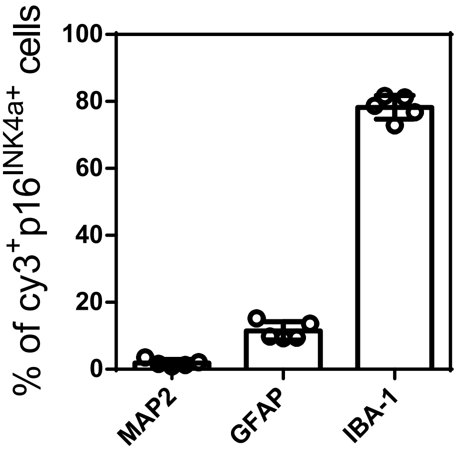

Figure S35. Detection of Cy3^{+} senescent neurons (MAP2^{+}), astrocytes (GFAP^{+}) and microglia (IBA-1^{+}) in the brain of PD mice after injection for 12 h by flow cytometry.

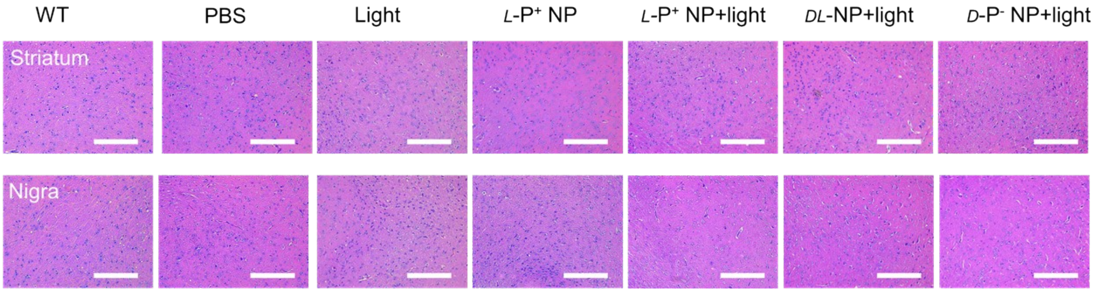

Figure S36. H&E staining assays of the striatum and nigra from mice with different treatments. Scale bars are 100 μm.

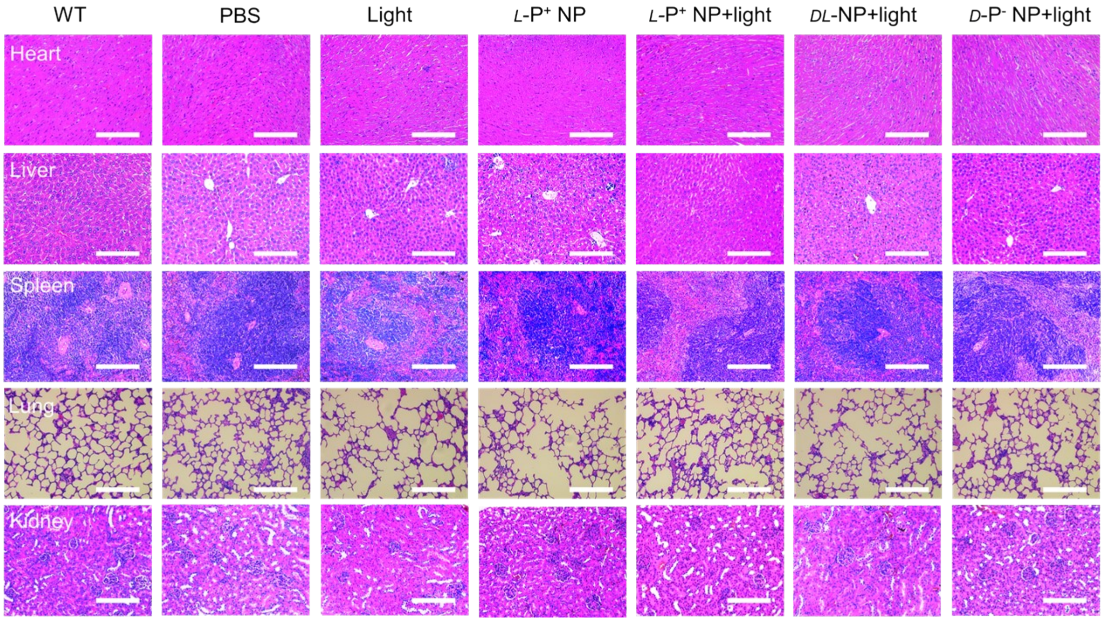

Figure S37. H&E staining assays of the heart, liver, kidney, spleen, lung from mice with different treatments. Scale bars are 100 μm.

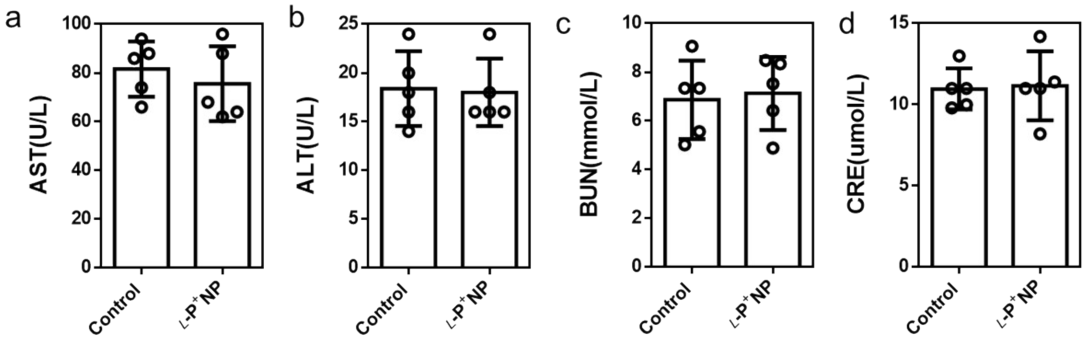

Figure S38. Serum biochemistry analysis of (**a**) AST, (**b**) ALT, (**c**) BUN and (**d**) CRE to reveal the biocompatibility of NPs in liver and kidney. Data are presented as mean ± s. d. (n = 5).

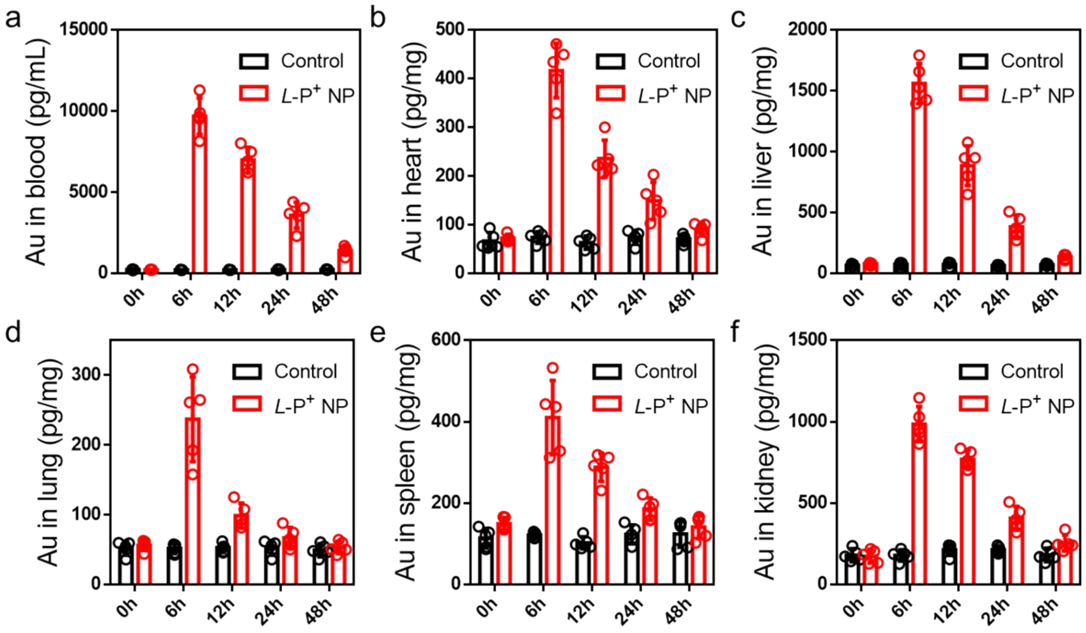

Figure S39. Biodistribution of NPs in major organs and blood for different time, (**a**) blood, (**b**) heart, (**c**) liver, (**d**) lung, (**e**) spleen and (**f**) kidney. Data are presented as mean ± s. d. (n = 5).

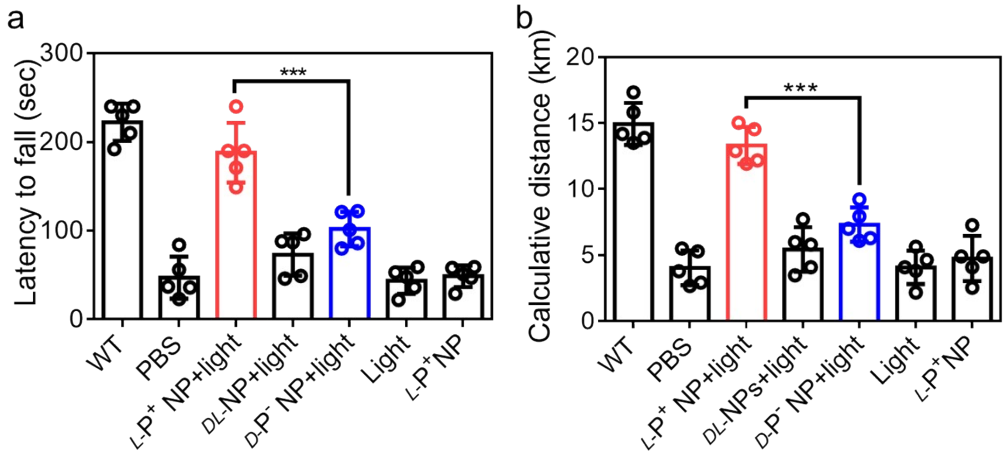

Figure S40. (**a**) The latency to fall of mice on rotarod treated with only light or *L*-P^+ NP in rotarod test. (**b**) Calculative distance of mice treated with light and *L*-P^+ NP in wheel running test for 60 min. Data are presented as mean ± s. d. (n = 5).

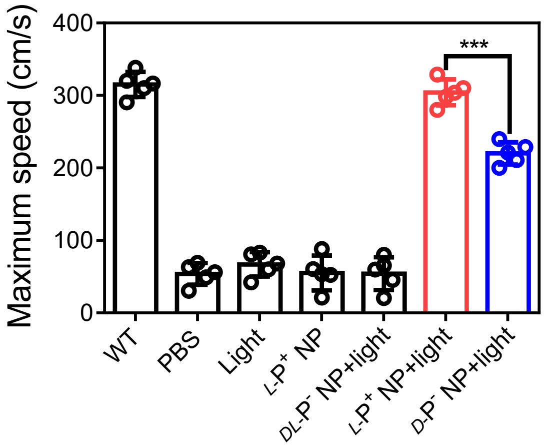

Figure S41. Maximum speed of mice with different treatments in wheel running test for 60 min. Data are presented as mean ± s. d. (n = 5). ***p<0.001.

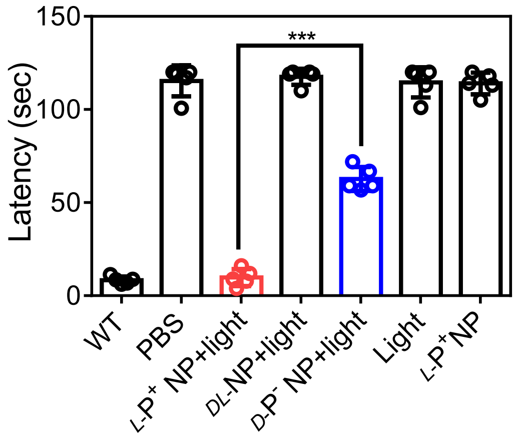

Figure S42. Latency for escape to platform in water maze experiment. Data are presented as mean ± s. d. (n = 5). ***p<0.001.

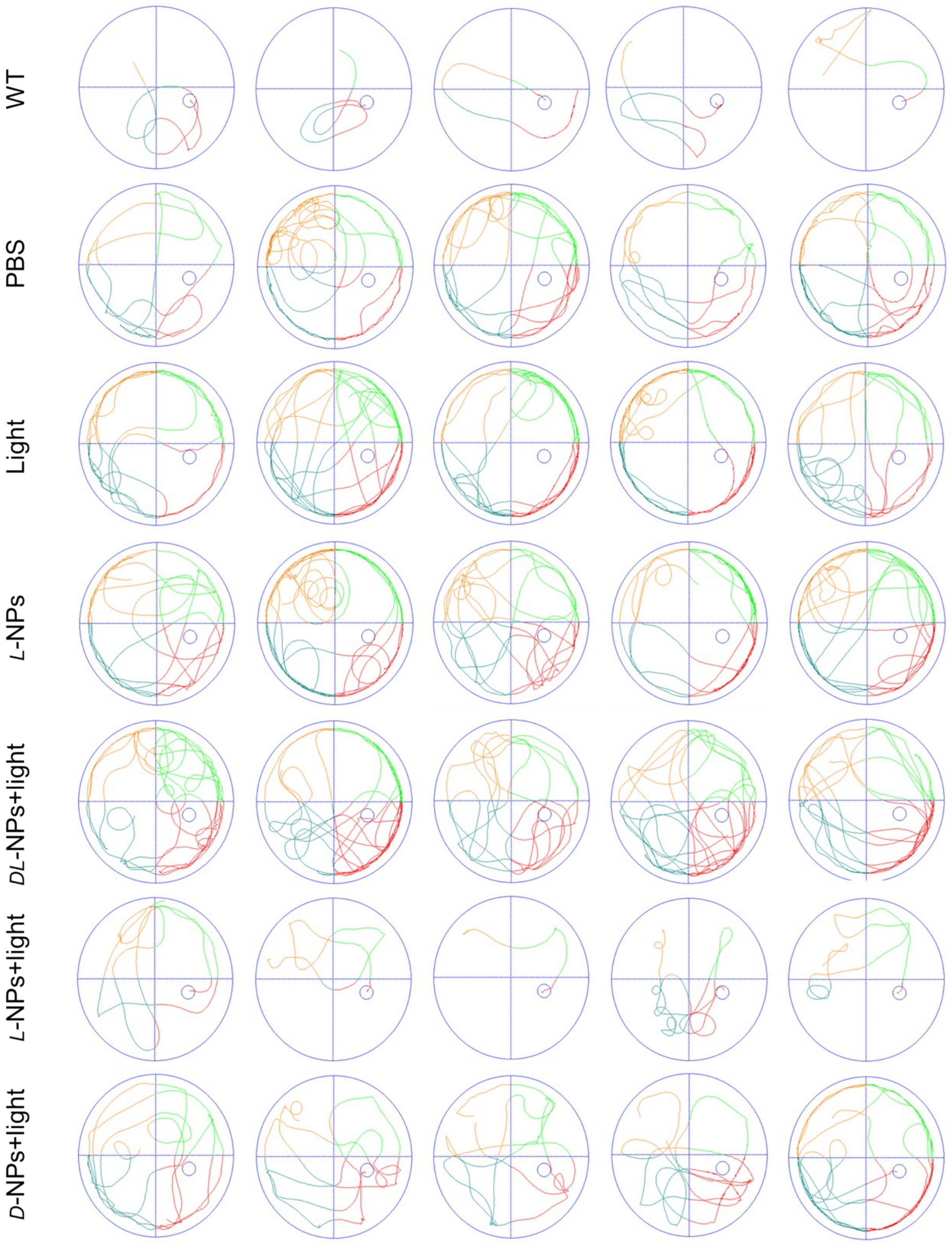

Figure S43. Tracks sheets show the alteration in the locomotion of different mice (*n* = 5).

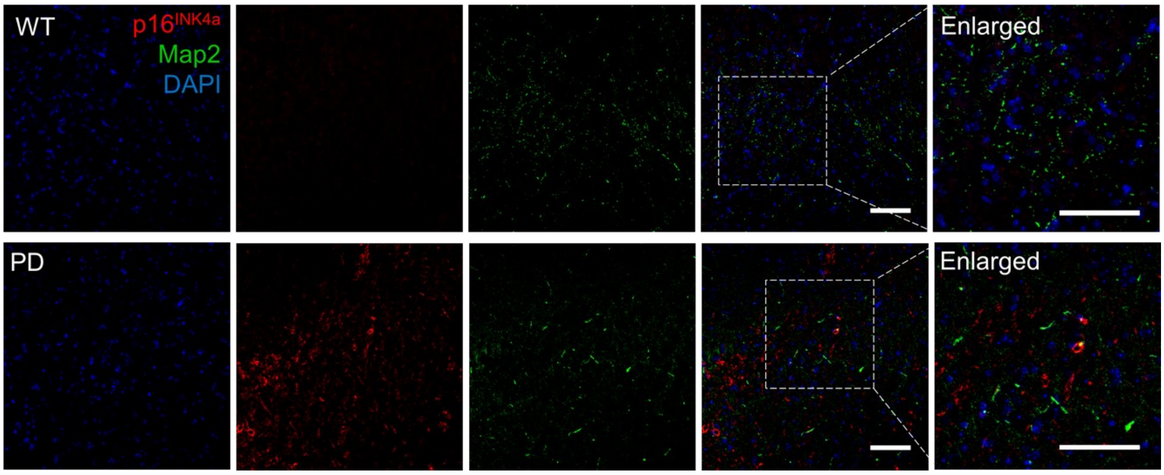

Figure S44. Confocal images of Map2 (green) and p16^{INK4a} (red) in sections of nigra from WT mice and PD mice. Scale bars, 60 μm.

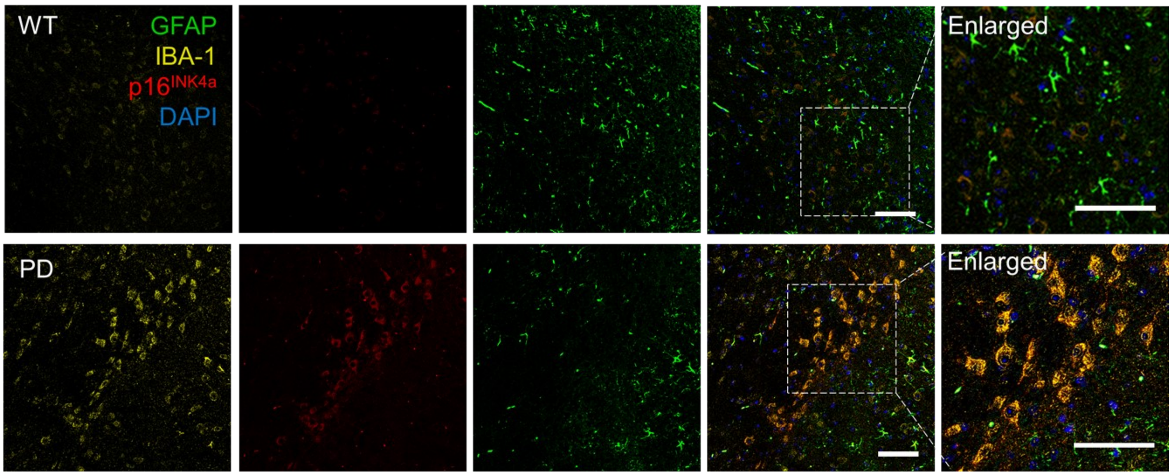

Figure S45. Confocal images of GFAP (green), IBA-1(yellow) and p16^{INK4a} (red) in sections of nigra from WT mice and PD mice. Scale bars, 60 μm.

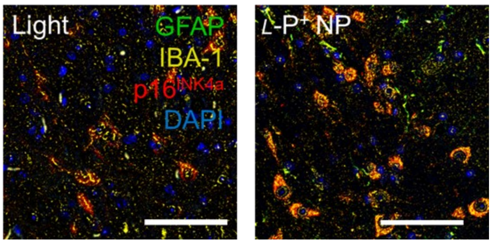

Figure S46. Confocal images of GFAP, IBA-1 and p16^{INK4a} in nigra of different mice. green, GFAP; yellow, IBA-1; blue, DAPI for nuclei. Scale bars, 60 μm.

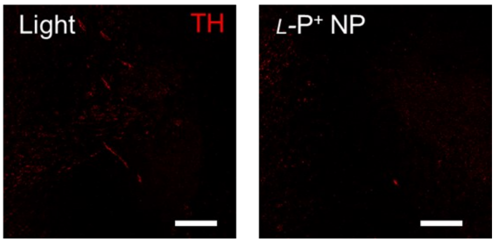

Figure S47. Confocal images TH in nigra of different mice. Scale bars, 50 μm.

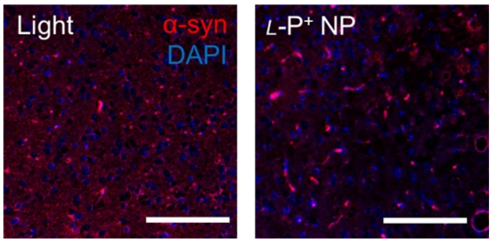

Figure S48. Confocal images α-syn in nigra of different mice. Scale bars, 100 μm.

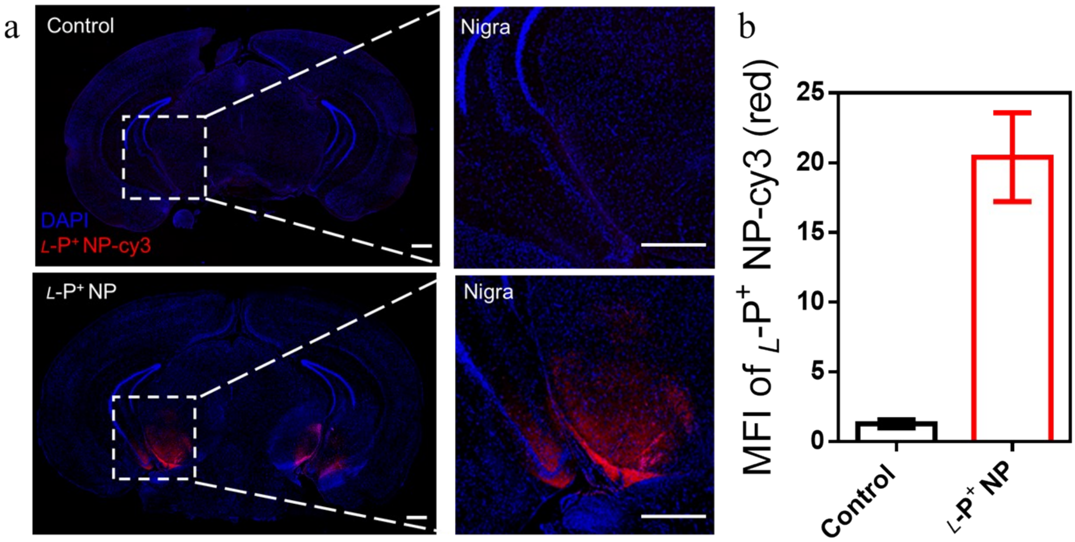

Figure S49. (**a**) IHF images of *L*-P^+ NP-Cy3 in brain of PD mice. PD mice without injection was set as control. Scale bars, 0.5 cm. (**b**) MFI of *L*-P^+ NP-Cy3 in nigra for the result in **Figure a.** Data are presented as mean ± s. d. (n = 5).

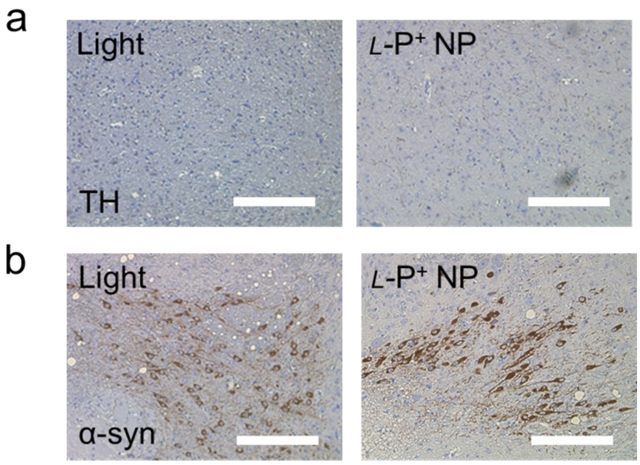

Figure S50. IHC images of (**a**) TH and (**b**) α-syn in the brains (substantia nigra) of mice treated with light and *L*-P$^+$ NP, respectively. Scale bars, 100 μm.

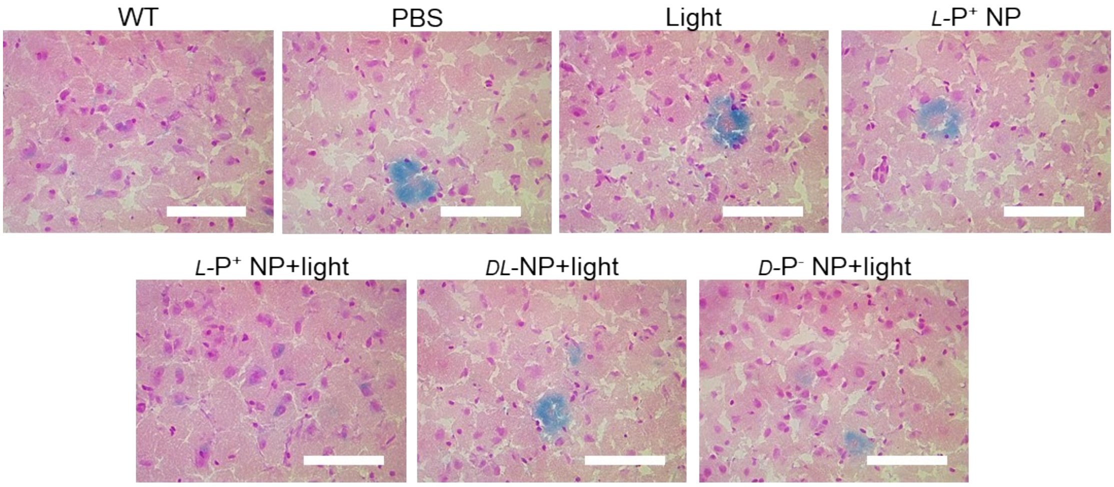

Figure S51. Representative images of β-gal staining of brain in nigra of different mice. Data are presented as mean ± s. d. (n = 5). Scale bars, 50 μm.

Reference

1. Xu, L.; Wang, X.; Wang, W.; Sun, M.; Choi, W. J.; Kim, J.-Y.; Hao, C.; Li, S.; Qu, A.; Lu, M.; Wu, X.; Colombari, F. M.; Gomes, W. R.; Blanco, A. L.; de Moura, A. F.; Guo, X.; Kuang, H.; Kotov, N. A.; Xu, C., Enantiomer-dependent immunological response to chiral nanoparticles. *Nature* **2022,** *601* (7893), 366-373.
